# The Association of Alcohol Consumption with Glaucoma and Related Traits: *Findings from the UK Biobank*

**DOI:** 10.1016/j.ogla.2022.11.008

**Published:** 2022-12-05

**Authors:** Kelsey V. Stuart, Robert N. Luben, Alasdair N. Warwick, Kian M. Madjedi, Praveen J. Patel, Mahantesh I. Biradar, Zihan Sun, Mark A. Chia, Louis R. Pasquale, Janey L. Wiggs, Jae H. Kang, Jihye Kim, Hugues Aschard, Jessica H. Tran, Marleen A.H. Lentjes, Paul J. Foster, Anthony P. Khawaja, on behalf of the Modifiable Risk Factors for Glaucoma Collaboration, the UK Biobank Eye and Vision Consortium, and the International Glaucoma Genetics Consortium, Members of the Modifiable Risk Factors for Glaucoma Collaboration, Members of the UK Biobank Eye and Vision Consortium, Members of the International Glaucoma Genetics Consortium

**Affiliations:** 1 NIHR Biomedical Research Centre, Moorfields Eye Hospital NHS Foundation Trust and UCL Institute of Ophthalmology, London, United Kingdom.; 2 MRC Epidemiology Unit, University of Cambridge, Cambridge, United Kingdom.; 3 UCL Institute of Cardiovascular Science, University College London, London, United Kingdom.; 4 Department of Ophthalmology, University of Calgary, Calgary, Alberta, Canada.; 5 Department of Ophthalmology, Icahn School of Medicine at Mount Sinai, New York, New York.; 6 Department of Ophthalmology, Massachusetts Eye and Ear, Harvard Medical School, Boston, Massachusetts.; 7 Department of Medicine, Brigham and Women’s Hospital, Harvard Medical School, Boston, Massachusetts.; 8 Department of Epidemiology, Harvard T. H. Chan School of Public Health, Boston, Massachusetts.; 9 Institut Pasteur, Université Paris Cité, Department of Computational Biology, Paris, France.; 10 School of Medical Sciences, Örebro University, Örebro, Sweden.

**Keywords:** Alcohol, Glaucoma, Intraocular pressure, OCT, UK Biobank

## Abstract

**Purpose:**

To examine the associations of alcohol consumption with glaucoma and related traits, to assess whether a genetic predisposition to glaucoma modified these associations, and to perform Mendelian randomization (MR) experiments to probe causal effects.

**Design:**

Cross-sectional observational and gene–environment interaction analyses in the UK Biobank. Two-sample MR experiments using summary statistics from large genetic consortia.

**Participants:**

UK Biobank participants with data on intraocular pressure (IOP) (n = 109 097), OCT-derived macular inner retinal layer thickness measures (n = 46 236) and glaucoma status (n = 173 407).

**Methods:**

Participants were categorized according to self-reported drinking behaviors. Quantitative estimates of alcohol intake were derived from touchscreen questionnaires and food composition tables. We performed a 2-step analysis, first comparing categories of alcohol consumption (never, infrequent, regular, and former drinkers) before assessing for a dose-response effect in regular drinkers only. Multivariable linear, logistic, and restricted cubic spline regression, adjusted for key sociodemographic, medical, anthropometric, and lifestyle factors, were used to examine associations. We assessed whether any association was modified by a multitrait glaucoma polygenic risk score. The inverse-variance weighted method was used for the main MR analyses.

**Main Outcome Measures:**

Intraocular pressure, macular retinal nerve fiber layer (mRNFL) thickness, macular ganglion cell–inner plexiform layer (mGCIPL) thickness, and prevalent glaucoma.

**Results:**

Compared with infrequent drinkers, regular drinkers had higher IOP (+0.17 mmHg; *P* < 0.001) and thinner mGCIPL (−0.17 μm; *P* = 0.049), whereas former drinkers had a higher prevalence of glaucoma (odds ratio, 1.53; *P* = 0.002). In regular drinkers, alcohol intake was adversely associated with all outcomes in a dose-dependent manner (all *P* < 0.001). Restricted cubic spline regression analyses suggested nonlinear associations, with apparent threshold effects at approximately 50 g (~6 UK or 4 US alcoholic units)/week for mRNFL and mGCIPL thickness. Significantly stronger alcohol–IOP associations were observed in participants at higher genetic susceptibility to glaucoma (*P*_interaction_ < 0.001). Mendelian randomization analyses provided evidence for a causal association with mGCIPL thickness.

**Conclusions:**

Alcohol intake was consistently and adversely associated with glaucoma and related traits, and at levels below current United Kingdom (< 112 g/week) and United States (women, < 98 g/week; men, < 196 g/week) guidelines. Although we cannot infer causality definitively, these results will be of interest to people with or at risk of glaucoma and their advising physicians.

**Financial Disclosure(s):**

Proprietary or commercial disclosure may be found after the references.

Alcohol consumption is a leading cause of death and disability worldwide, responsible for an estimated 3 million deaths and 132 million disability-adjusted life years lost in 2016 alone.^1,2^ Alcohol use has been implicated in over 200 diverse health conditions, and it therefore represents a significant public health concern and an important modifiable lifestyle risk factor.^2^ Despite these well-documented harms, it remains a highly prevalent behavior in many populations and particularly in Europe, where 60% of all adults are reported to be current alcohol drinkers.^2^

Intraocular pressure (IOP) remains the major modifiable risk factor for glaucoma, but there is considerable interest in identifying other factors that may complement existing treatment strategies or guide lifestyle recommendations. Given the widespread prevalence of both alcohol consumption and glaucoma, an understanding of the magnitude and shape of any underlying association may have important clinical and public health consequences.

The acute ophthalmic effects of alcohol consumption include transient ocular hypotension and an increase in blood flow to the optic nerve head, theoretically playing a protective role in the development of glaucoma.^3–5^ However, alcohol has known neurotoxic properties, and chronic use has been associated with multiple neurodegenerative conditions, which may have similar implications for glaucoma risk.^6^ Previous studies of the association between alcohol consumption and glaucoma have failed to yield consistent results, and although a recent systematic review and meta-analysis has suggested that habitual alcohol use is adversely associated with both IOP and open-angle glaucoma, firm conclusions are limited by marked heterogeneity and a high risk of bias.^7^

Observational studies of alcohol and glaucoma should be adequately powered to detect an association despite noise in the assessment variables, allow for quantification of alcohol intake to explore possible dose-response and nonlinear relationships, adjust for key covariates to limit residual confounding, and assess relationships with a variety of glaucoma-related traits to gauge the consistency of any observed associations. Additionally, the availability of genetic data would allow for consideration to be given to gene–environment interactions and causal effects through Mendelian randomization (MR) experiments.

The UK Biobank fulfills all the aforementioned criteria and represents an invaluable resource that may be lever-aged to further our understanding of the alcohol–glaucoma relationship. We utilized UK Biobank questionnaire, anthropometric, ocular, medical, and lifestyle data to explore the association of alcohol consumption with glaucoma and various glaucoma-related traits. We also used genetic data to consider possible modification of the alcohol–glaucoma association by a glaucoma polygenic risk score (PRS) and performed 2-sample MR experiments using summary statistics from large genome-wide association studies (GWASs) to probe causal effects.

## Methods

### UK Biobank

The UK Biobank is a large, population-based cohort study of over half a million participants aged 37 to 73 years at baseline (2006–2010). Participants were recruited through National Health Service registers and invited to attend one of 22 assessment centers across the United Kingdom where extensive phenotypic information and biological samples were collected.^8,9^ After providing electronic informed consent, participants completed an in-depth touchscreen questionnaire—detailing sociodemographic information, life-course exposures, and medical history—and an array of physical and cognitive measurements. Blood, urine, and saliva specimens were also collected and used to generate a wealth of genetic, proteomic, and metabolomic data.^10^ Multiple repeat and supplementary assessments, including an eye and vision substudy (performed in 2009–2010), have been conducted in participant subsets to augment the baseline data.^11^ Additional health-related outcomes are available through linkage with nationwide health records and registries. Detailed descriptions of the UK Biobank, including the overall study protocol and individual test procedures are available online (https://www.ukbiobank.ac.uk). The UK Biobank was approved by the National Health Service North West Multicentre Research Ethics Committee (06/MRE08/65) and the National Information Governance Board for Health and Social Care. This research was conducted under UK Biobank application number 36741 and conformed to the tenets of the Declaration of Helsinki.

### Assessment and Quantification of Alcohol Intake

Information on habitual alcohol consumption was assessed in the baseline questionnaire (2006–2010). Participants were asked how often they drank alcohol and were required to categorize their response as: “Daily/almost daily,” “3–4 times a week,” “1–2 times a week,” “1–3 times a month,” “Special occasions only,” or “Never.” If their alcohol consumption varied substantially, participants were asked to provide an average considering their intake over the last year. Participants who reported a drinking frequency of “1–2 times a week” or greater were then asked to quantify their average weekly alcohol intake, whereas those reporting a frequency of “1–3 times a month” or “Special occasions only” were asked about their average monthly intake of each of the following: (1) “Glasses of red wine”; (2) “Glasses of white wine or champagne”; (3) “Pints of beer or cider”; (4) “Measures of spirits or liquors”; (5) “Glasses of fortified wine”; and (6) “Glasses of other alcoholic drinks.” These questions included definitions, examples, and standard portion sizes for each of the 6 alcoholic beverage types. Participants who reported a drinking frequency of “Never” to the first question were not asked to quantify their alcohol intake but were asked if they had previously drunk alcohol. Participants were additionally asked whether they usually consumed alcohol with meals.

For the purposes of this study, participants were categorized as never drinkers (frequency = “Never”; previously drunk alcohol = “No”), infrequent drinkers (frequency = “Special occasions only”), regular drinkers (frequency = “1–3 times a month” or greater), or former drinkers (frequency = “Never”; previously drunk alcohol = “Yes”).

We then calculated average total alcohol (ethanol) intake (g/week) for all regular drinkers according to the formula:

$$\sum_{i=1}^{6}{\text{number of portions}}_{\left(i\right)}\times \text{portion size}{\left(\text{mL}\right)}_{\left(i\right)}\times \text{alcohol concentration}{\left(\text{g}/\text{mL}\right)}_{\left(i\right)}\times k$$

where i represents the alcoholic beverage categories described above and k represents a conversion factor depending on whether an individual reported their average weekly (k = 1) or monthly (k = 0.23) alcohol intake. For those reporting a weekly intake, the conversion factor does not change the quantitative estimate, whereas for those reporting a monthly intake, the conversion factor represents: (× 12 months/365 days × 7 days).

The alcohol concentrations applied to each alcoholic beverage category were based on the same food composition tables and methodology used for the Oxford WebQ, a validated web-based food frequency questionnaire that has been used to calculate alcohol intake in UK Biobank 24-hour dietary follow-up assessments.^12–14^ To handle implausibly low (e.g., regular drinkers reporting a weekly intake of 0 g) and extreme upper values, we excluded total alcohol intake estimates in the top and bottom 1 percentiles. Further details of the derivation of alcohol intake from the UK Biobank baseline questionnaire are available in Figure S1 and Table S1 (available at www.ophthalmologyglaucoma.org).

### Glaucoma-Related Outcome Measures and Case Ascertainment

The UK Biobank outcomes utilized in this study were IOP, 2 OCT-derived macular inner retinal thickness measures, and prevalent glaucoma status. All outcomes were assessed on the same day as the alcohol assessment.

#### Intraocular pressure:

In 2009–2010, IOP measurements in both eyes of ~115 000 participants were taken using an Ocular Response Analyzer non-contact pneumotonometer (Reichert Corp).^11^ Participants reporting an eye infection or eye surgery within the previous 4 weeks did not undergo IOP assessment. Individual-level IOP values were calculated as the mean of available right and left eye values, and extreme IOP values in the top and bottom 0.5 percentiles were excluded. For this analysis, we used corneal-compensated IOP, a measure derived from a linear combination of inward and outward applanation tensions, which is least influenced by corneal biomechanical properties.^15^ We excluded participants with a history of glaucoma surgery or laser therapy, corneal graft, or refractive surgery or visually-significant ocular trauma (these participants were not excluded from the analyses of OCT parameters or glaucoma status). We imputed pretreatment IOP values for participants using ocular hypotensive agents by dividing the measured IOP by 0.7, as previously described.^16^

#### OCT:

In 2009 to 2010, macular spectral domain OCT imaging using a Topcon 3D OCT-1000 Mark II (Topcon Corp) was performed in both eyes of ~65 000 participants.^11^ The image handling, segmentation and quality control protocols have been described previously.^17^ For this analysis, we used macular retinal nerve fiber layer (mRNFL) thickness and macular ganglion cell–inner plexiform layer (mGCIPL) thickness, as these measures have been shown to be useful glaucoma-related biomarkers.^18,19^ We calculated individual-level OCT values as the mean of all available right and left eye measurements. As we aimed to explore associations in the general population, we did not exclude individuals with retinal (or other) pathology from the OCT analyses.

#### Glaucoma status:

From 2006 to 2010, the touchscreen questionnaire administered to ~175 000 participants included a question on physician-diagnosed eye disorders. Participants were considered cases if they reported a diagnosis of glaucoma, or previous surgical or laser treatment for glaucoma, in either eye. We also included any participant carrying an *International Classification of Diseases* (ICD) code for glaucoma (ICD9: 365.* [excluding 365.0]; ICD10: H40.* [excluding H40.0 and H42.*]) in their linked hospital records at any point before and up to 1 year after the baseline assessment. We excluded cases who were diagnosed before 30 years of age and controls who reported using ocular hypotensive medication or carrying an ICD code for glaucoma suspect (ICD9: 365.0; ICD10: H40.0).

### Genotyping and PRSs

Genetic data for ~490 000 participants were generated using 2 closely related genotyping platforms. The Affymetrix UK BiLEVE Axiom Array returned genotypes at 807 411 markers for ~50 000 participants, whereas the Affymetrix UK Biobank Axiom Array provided genotypes at 825 925 markers for the remaining ~440 000 participants.^20^ Quality control and imputation were performed jointly for these 2 platforms, as previously described.^9^ Imputation (genotypic determination based on inference and not by direct typing) was based on the UK10K and Haplotype Reference Consortium reference panels.

To assess whether observed exposure–outcome associations were modified by genetic factors (gene–environment interaction), we constructed a PRS based on 2 673 independent single nucleotide polymorphisms (SNPs) associated with glaucoma (at *P* ≤ 0.001) from a recent multitrait analysis of GWASs (MTAG) which included UK Biobank data.^21^ Glaucoma is a complex polygenic disease, and we considered the MTAG PRS to be a better representation of genetic variation in glaucoma than any individual or limited set of variants. We used the effect estimates from the original MTAG study to generate a glaucoma PRS for each participant using a standard weighted sum of individual SNPs:

$$\sum_{i=1}^{2\;673}{\hat{\beta }}_{\left(i\right)}\times SNP_{\left(i\right)}$$

Where, ${\hat{\beta }}_{\left(i\right)}$ is the estimated effect size of ${SNP}_{\left(i\right)}$ on glaucoma. The PRS was normalized with a mean of 0 and a standard deviation (SD) of 1 for analyses. This glaucoma MTAG PRS has been found to be predictive of earlier age at glaucoma diagnosis, glaucoma progression, and need for surgical intervention in an independent cohort.^21^

### Statistical Analysis

Baseline characteristics for each cohort (IOP, OCT, and glaucoma) and according to alcohol drinking status were summarized as mean (SD) for continuous variables, and frequency (proportion, %) for categorical variables. Alcohol intake demonstrated a right-skewed distribution, and these data were summarized as median (interquartile range).

To assess the main associations between alcohol intake and the various glaucoma-related outcomes, we used multivariable linear (for IOP, mRNFL thickness, and mGCIPL thickness) and logistic (for glaucoma) regression models adjusted for key sociodemographic, medical, anthropometric, ocular, and lifestyle factors. We included the following covariates (all of which were ascertained on the same day as the alcohol and ophthalmic assessments) based on previously reported risk factors and associations^22^: age (years), sex (women, men), self-reported ethnicity (White, Black, and Other), Townsend deprivation index (a measure of material deprivation based on an individual’s residential postcode; a higher index score indicates greater relative poverty), body mass index (kg/m^2^; calculated as weight/height^2^), height (cm), systolic blood pressure (mmHg; calculated as the mean of 2 measurements), spherical equivalent (diopters; calculated as spherical power + one-half cylindrical power; the mean of all available right and left eye values were used for this analysis), self-reported diabetes mellitus, smoking status (never, previous, and current), smoking intensity (cigarettes per day; never and previous smokers were assigned a value of 0), physical activity (metabolic equivalent of task minutes/week; a measure of total energy expenditure based on an adapted version of the validated International Physical Activity Questionnaire),^23^ and assessment season (Summer, Autumn, Winter, or Spring).

We first assessed associations in all available participants according to alcohol intake category. In epidemiological studies of alcohol intake, the use of low volume drinkers as the reference group offers several advantages compared with the use of never drinkers.^24^ We therefore used infrequent drinkers as the reference category for this step of the analysis. Subsequent quantitative analyses were then restricted to regular drinkers only, as the inclusion of never and former drinkers, who tend to differ substantively from current drinkers, may introduce bias.^25^ Additionally, because infrequent drinkers (who by definition consumed alcohol less than once a month) were asked to quantify their monthly alcohol intake, we deemed estimates of their alcohol intake less accurate than for regular drinkers, and these participants were also excluded from subsequent analyses.

In the second step of our analysis, we aimed to assess for dose-response and nonlinear associations. For the dose-response analyses, alcohol intake was analyzed as both a continuous (g/week) and categorical (quintiles of alcohol intake) variable. Trends across quintiles were examined by testing the median value of each group. Nonlinear associations were assessed with restricted cubic spline regression models adjusted for the same covariates listed above. For each association, we considered 3 to 7 knots at fixed heuristic percentiles, as suggested by Harrell,^26^ with final model selection based on minimization of the Akaike Information Criterion. We used the natural logarithm of alcohol intake in these models, as this transformed variable was approximately normally distributed and aided graphical visualization of inflection points occurring at relatively low quantities of alcohol intake.

We conducted the following sensitivity analyses: (1) sex-stratified analyses with tests for interaction; (2) analyses restricted to participants of European descent only; (3) analyses according to alcohol beverage type; (4) interaction analyses to assess whether associations were modified by frequency of alcohol consumption or drinking alcohol with meals; (5) exclusion of participants with glaucoma for analyses of IOP and OCT parameters; (6) analyses using different definitions for glaucoma (ICD10 codes limited to primary open-angle glaucoma and undefined glaucoma); (7) analyses using different IOP measurements (Goldmann-correlated IOP and corneal-compensated IOP without imputation of pretreatment values); (8) analyses restricted to participants without hypertension (self-report or systolic blood pressure ≥ 140 mmHg); and (9) analyses including additional covariates in the final regression models—caffeine intake (mg/day), total cholesterol (mmol/L), statin use, and oral β-blocker use—based on recent results from similar analyses of glaucoma-related traits.^27–29^

To assess whether the relationship between alcohol intake and the various glaucoma-related traits were modified by the glaucoma MTAG PRS, we tested the significance of a multiplicative interaction term between alcohol intake and the genetic factor in the maximally adjusted regression models. The glaucoma MTAG PRS was included as a continuous variable in these models. Although UK Biobank participants were included in the original MTAG study from which the PRS weights were derived,^21^ the independence of marginal and interaction effects in these models limits the risk of data overfitting.

### MR Analyses

We assessed the possibility of causal effects of alcohol intake on glaucoma and related traits by performing MR analyses. MR is an instrumental variable (IV) approach, which allows for the evaluation of the association between a genetically determined risk factor (in this case, a genetic predisposition to higher alcohol consumption) and a particular trait or disease outcome.^30^ By leveraging the random allocation of alleles at conception, MR is analogous to a naturally occurring randomized control trial, which is less prone to confounding, reverse causation, and other biases than traditional epidemiological methods and, providing certain assumptions are satisfied, assists with inferring causal relationships.^30,31^ The IV comprises multiple genetic variants robustly associated with the risk factor of interest and captures an individual’s lifetime average exposure in a dose-response manner.

The rs1229984 variant in the alcohol dehydrogenase 1B (*ADH1B*) gene region is consistently and strongly associated with lower alcohol intake in European populations.^32–34^ Alcohol consumption in the presence of this genetic variant, however, leads to rapid accumulation of toxic intermediate metabolites and it is therefore also associated with higher levels of alcohol-related tissue damage.^33^ Given these biological associations, the inclusion of this SNP in an IV may bias MR results. We therefore considered 2 alcohol intake IVs in our analyses: a full instrument, comprised of all genetic variants from the GWAS & Sequencing Consortium of Alcohol and Nicotine use GWAS including rs1229984, and a restricted instrument, comprising the same variants but excluding rs1229984.^35^

We performed 2-sample MR analyses, in which the IV-exposure and IV-outcome associations are measured in different study populations, using summary-level data for European participants from published GWASs, as this design can provide substantially increased statistical power by combining data from multiple sources, including large consortia.^30^ The construction of our alcohol IV was based on results from the most recent GWAS of alcohol intake from the GWAS & Sequencing Consortium of Alcohol and Nicotine use (n = 941 280).^35^ For the outcomes, we utilized data from the largest available GWAS meta-analyses for IOP (n = 139 555)^16^ and primary open-angle glaucoma (n = 216 257), as well as GWAS results for mRNFL thickness and mGCIPL thickness based on UK Biobank participants of European descent with high-quality imaging and genotype data (n = 31 434).^36,37^ We additionally included a GWAS meta-analysis from the International Glaucoma Genetics Consortium for vertical cup disc ratio (vCDR) based on scanning laser ophthalmoscopy or optic disc photography (n = 23 899).^38^ MR analyses were conducted in accordance with the Strengthening the Reporting of Observational Studies in Epidemiology-Mendelian Randomization guidelines.^39^ Full details of the MR analyses are available in the Appendix (available at www.ophthalmologyglaucoma.org).

## Results

### Participants

**Figure 2.**
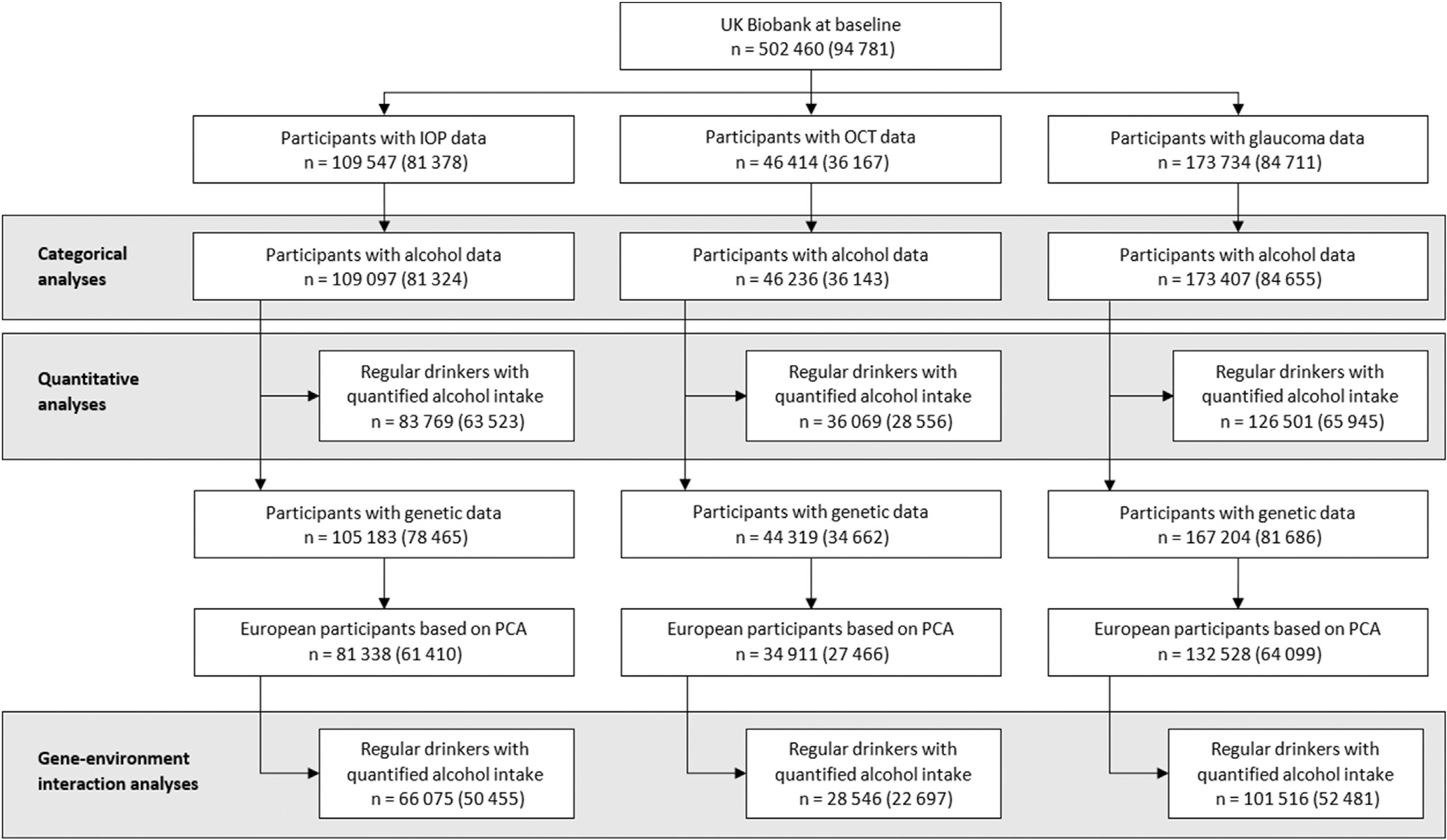
Flow diagram outlining eligible UK Biobank participants available for this study. Numbers in parentheses indicate participants with complete data for all covariables. IOP = intraocular pressure; PCA = principal components analysis.

The number of UK Biobank participants eligible for and included in each of our analyses is presented in Figure 2. Overall, we included 81 324, 36 143, and 84 655 participants with complete data for the analyses of IOP, OCT-derived macular inner retinal thickness measures, and glaucoma status, respectively. Participant characteristics for each of the 3 cohorts are summarized in Table 2. As there was considerable overlap across cohorts, demographic features and baseline characteristics were largely similar. In keeping with the overall UK Biobank, mean age was 56–57 years, with a slight female predominance (52%–53%) and a majority of White participants (90%–92%).

### Alcohol Intake

Overall, 80%–81% of participants were classified as regular drinkers, with a median alcohol intake of slightly more than 90 g/week. Among these participants, women were more likely to be red wine (38%) or white wine (29%–30%) drinkers, whereas men were more likely to be beer/cider (44%) or red wine (24%) drinkers. By contrast, infrequent drinkers comprised only 12% of participants, with a median alcohol intake of less than 3 g/week. Only 4%–5% and 4% of the cohort were classified as never and former drinkers, respectively. The distribution of alcohol intake among regular drinkers and stratified by sex is displayed in Figure S3 (available at www.ophthalmologyglaucoma.org). Further details of alcohol consumption according to cohort and sex are available in Table S3 (available at www.ophthalmologyglaucoma.org). Participant characteristics according to alcohol consumption category and quintile of alcohol intake for the glaucoma cohort (the largest of the 3 cohorts) are presented in Table S4 (available at www.ophthalmologyglaucoma.org). Crude average IOP, mRNFL thickness, mGCIPL thickness, as well as glaucoma prevalence according to the same categories are presented in Table S5 (available at www.ophthalmologyglaucoma.org).

Total alcohol intake demonstrated strong associations with known alcohol-associated biochemical parameters, including high-density lipoprotein cholesterol and mean corpuscular volume, after adjustment for all covariates used in the main analyses (both *P* < 0.001).^40^

### Categorical Analyses

In the maximally adjusted multivariable linear and logistic regression models (Table 6), when compared with infrequent drinkers, regular drinkers had higher IOP (0.17 mmHg; 95% confidence interval [CI], 0.10–0.24; *P*<0.001) and thinner mGCIPL (−0.17 μm; 95% CI, −0.33 to 0.00; *P* = 0.049), but no difference in mRNFL thickness (−0.10 μm; 95% CI, −0.23 to 0.02; *P* = 0.11), or prevalence of glaucoma (odds ratio [OR], 1.13; 95% CI, 0.95–1.34; *P* = 0.16). Former drinkers had a higher prevalence of glaucoma (OR, 1.53; 95% CI, 1.16–2.02; *P* = 0.002) and, interestingly, lower IOP (−0.15 mmHg; 95% CI, −0.28 to −0.01; *P* = 0.03). These results were materially unchanged when combining never and infrequent drinkers as the reference category.

### Quantitative Analyses

When considering regular drinkers only, consistent linear dose-response relationships between alcohol intake and all of the glaucoma-related outcomes were observed. Each additional SD increase in alcohol intake (111–112 g/week) was associated with higher IOP (0.08 mmHg; 95% CI, 0.05–0.11), thinner mRNFL (−0.17 μm; 95% CI, −0.22 to −0.12), thinner mGCIPL (−0.34 μm; 95% CI, −0.40 to −0.27), and higher prevalence of glaucoma (OR, 1.11; 95% CI, 1.05–1.18) (all *P* < 0.001). Similarly, when compared with the lowest alcohol intake quintile (median 18–19 g/week), those in the highest alcohol intake quintile (median 278–280 g/week) had higher IOP (0.27 mmHg; 95% CI, 0.19–0.36), thinner mRNFL (−0.41 μm; 95% CI, −0.56 to −0.27), thinner mGCIPL (−0.83 μm; 95% CI, −1.02 to −0.63), and higher prevalence of glaucoma (OR, 1.36; 95% CI, 1.12–1.66) (all *P*_*trend*_ ≤ 0.001). Full details of the main analyses are presented in Table 6.

**Figure 4.**
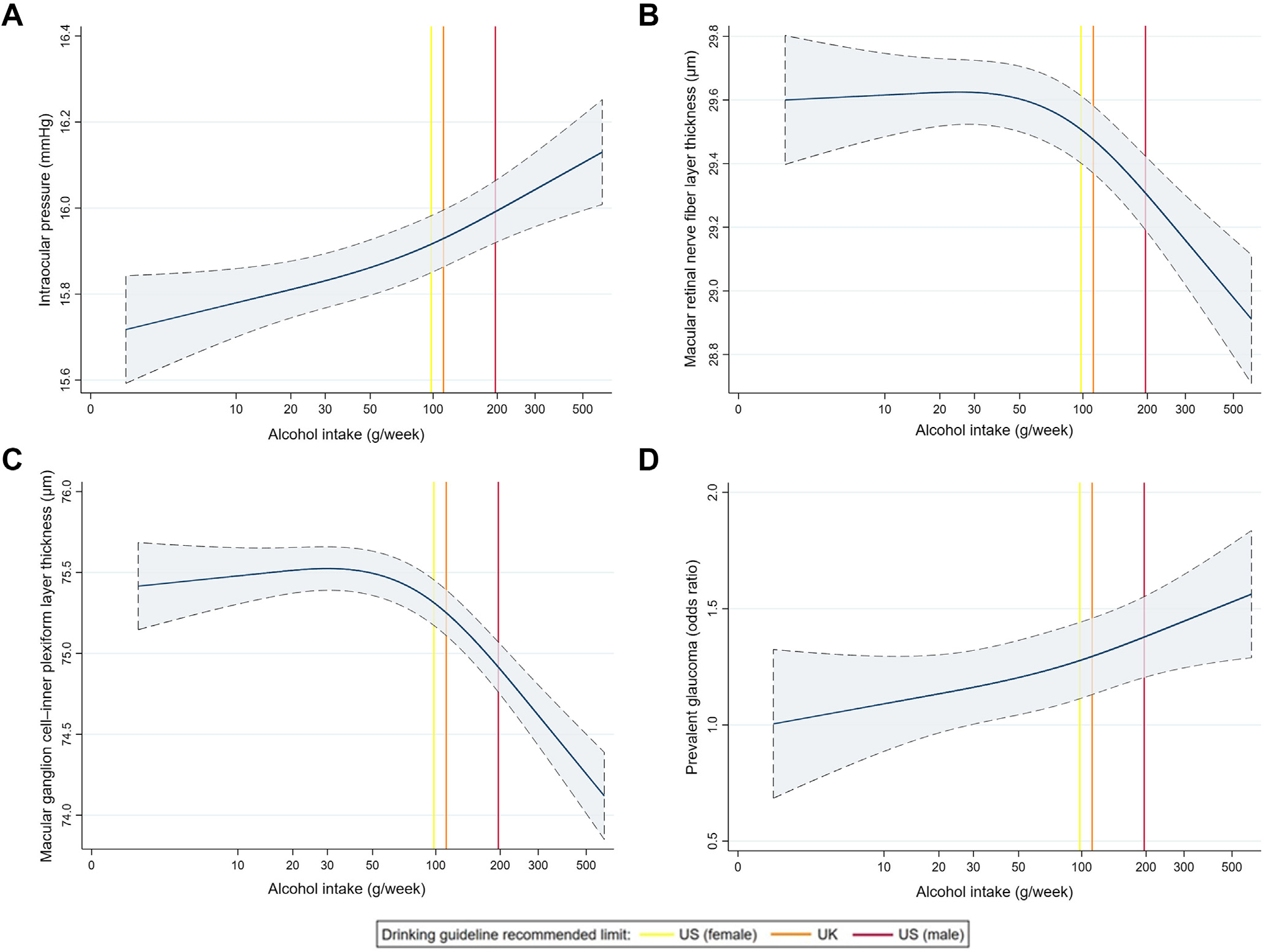
Maximally adjusted restricted cubic spline regression models for the association between alcohol intake and **A**, intraocular pressure; **B**, macular retinal nerve fiber layer thickness; **C**, macular ganglion cell–inner plexiform layer thickness; and **D**, glaucoma in regular drinkers. Vertical lines represent current UK (112 g/week) and US (women 98 g/week; men 196 g/week) recommended alcohol drinking guidelines.^41,42^

Maximally adjusted restricted cubic spline regression models suggested the presence of nonlinear associations (Fig 4). Although there was a clear log–linear relationship with IOP and glaucoma, there appeared to be a threshold effect of the log of alcohol intake on mRNFL thickness and mGCIPL thickness, with adverse associations only apparent after approximately 50 g (approximately 6 UK or 4 US alcoholic units)/week. The same threshold effect on the inner retinal OCT parameters was apparent when modeling associations with an untransformed alcohol intake variable. Importantly, adverse associations with all glaucoma-related outcomes were demonstrated at quantities below current recommended UK (<112 g/week) and US (women <98 g/week; men <196 g/week) drinking guidelines.^41,42^ When including all participants, with the exception of former drinkers, in these analyses (never drinkers were assigned an alcohol intake of 0 g/week), a similar threshold effect was additionally observed for glaucoma, but not for IOP (Fig S5, available at www.ophthalmologyglaucoma.org). Full details of the restricted cubic spline regression analyses and model selection are available in Table S7 (available at www.ophthalmologyglaucoma.org).

### Sensitivity Analyses

There was no evidence for a differential effect or interaction by sex (Tables S8 and S9, available at www.ophthalmologyglaucoma.org). Results were materially unchanged when restricting analyses to participants of European descent or those without hypertension. Results were generally consistent across all alcoholic beverage types (Table S10, available at www.ophthalmologyglaucoma.org), and there was no evidence for interaction according to frequency of alcohol consumption or drinking alcohol with meals. Exclusion of participants with glaucoma and the use of different glaucoma definitions did not yield different results, and similarly, results were largely unchanged when using different IOP definitions, although larger effect sizes and a null IOP association with former drinkers were noted with Goldmann-correlated IOP (Table S11, available at www.ophthalmologyglaucoma.org). The inclusion of additional covariables did not materially change the results, although there was a loss of statistical power because of fewer participants with complete data (Table S12, available at www.ophthalmologyglaucoma.org).

### Gene–Environment Interaction Analyses

**Figure 6.**
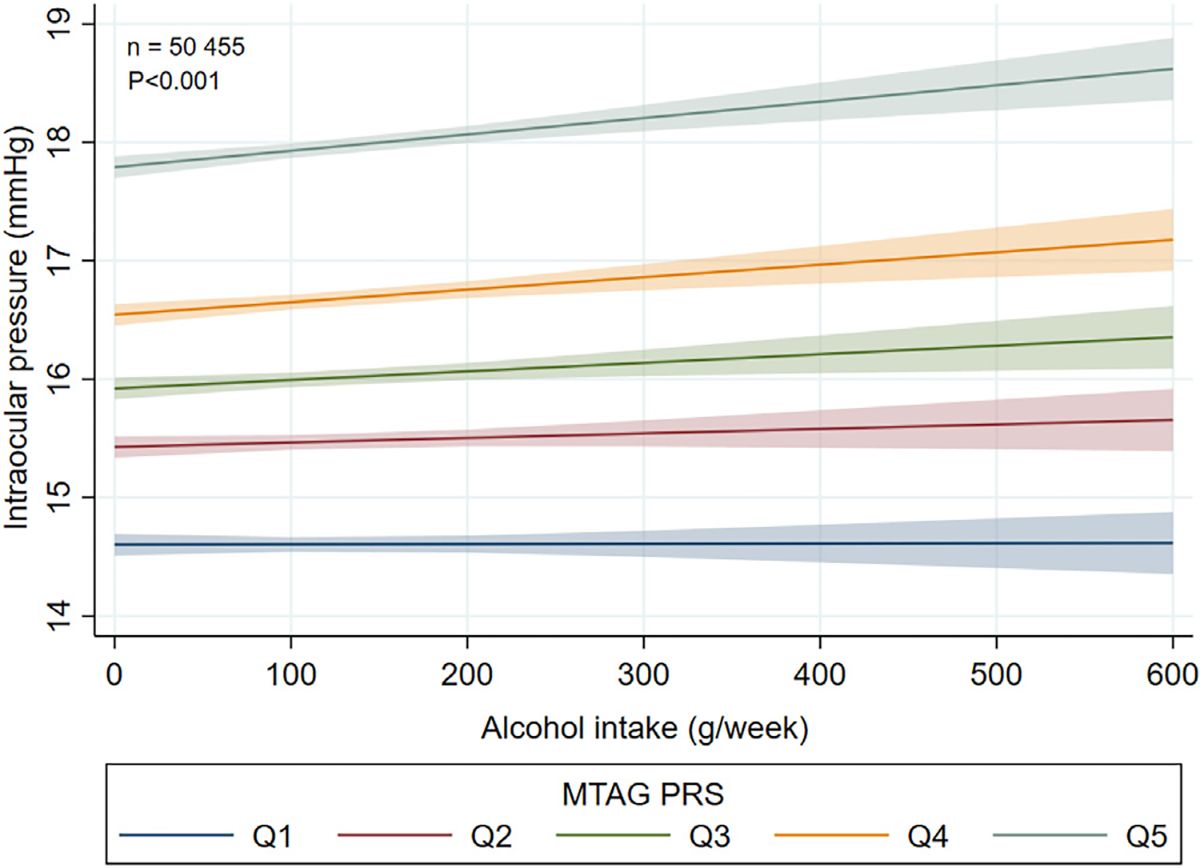
Gene–environment interaction analysis for the effect of the glaucoma MTAG PRS on the association between alcohol intake and intraocular pressure in regular drinkers of European ancestry. MTAG = multitrait analysis of genome-wide association studies; PRS = polygenic risk score; Q = quintile.

The glaucoma MTAG PRS was found to significantly modify the association between alcohol intake and IOP (*P*_interaction_ < 0.001), but not mRNFL, mGCIPL, or glaucoma (all *P* ≥ 0.21). No association with alcohol intake was observed in participants in the lowest quintile of genetic risk, with progressively stronger associations noted in subsequent quintiles (Fig 6). Specifically, for those in the highest glaucoma MTAG PRS quintile, each SD increase in alcohol intake was associated with 0.15 mmHg (95% CI, 0.07–0.24) higher IOP, compared with 0.00 mmHg (95% CI, −0.06 to 0.06), 0.04 mmHg (95% CI, −0.04 to 0.12), 0.08 mmHg (95% CI, −0.01 to 0.16), and 0.11 mmHg (95% CI, 0.03–0.20) for those in quintiles 1 to 4, respectively.

### MR Analyses

Inverse-variance weighted (IVW) MR experiments using the full alcohol genetic instrument (all genetic variants, including rs1229984) provided evidence for a causal effect of alcohol intake on mGCIPL thickness (−1.52 μm per SD increase in the instrument; 95% CI, −2.55 to −0.50; *P* = 0.004) but not IOP, mRNFL thickness, vCDR, or primary open-angle glaucoma (all *P* ≥ 0.13). The main mGCIPL result was supported by both the MR-PRESSO (MR pleiotropy residual sum and outlier) and multivariable MR methods (Table 13).

Similar MR experiments using the restricted alcohol instrument (all genetic variants, excluding rs1229984) provided stronger evidence for a causal association with mGCIPL, with a stronger IVW estimate (−2.07 μm per SD increase in the instrument; 95% CI, −3.22 to −0.93; *P* < 0.001) and consistent, generally significant, results across all alternative MR methods (Table 13). Additionally, this approach provided weak evidence for a causal association with mRNFL thickness, with a marginally significant IVW estimate (−0.98 μm per SD increase in the instrument; 95% CI, −1.89 to −0.07; *P* = 0.04) and consistent, albeit insignificant, estimates across all alternative MR methods. Although there was no evidence for a causal relationship with vCDR under the IVW method, multivariable MR yielded a marginally significant result (0.03 increase in vCDR per SD increase in the instrument; 95% CI, 0.00–0.06; *P* = 0.03).

With respect to the mGCIPL estimates, despite evidence for global heterogeneity for both the full and restricted alcohol instruments (Cochran’s *Q* statistic, *P* = 0.02 and *P* = 0.04, respectively), the MR-Egger intercept test did not suggest average directional pleiotropy (*P* = 0.06 and *P* = 0.55, respectively). Full results of the MR analyses, including SNP details, scatter plots, tests of heterogeneity, directional pleiotropy, and regression dilution statistics are available in the Appendix (available at https://www.ophthalmologyglaucoma.org).

## Discussion

In this study, we utilized data from the UK Biobank and multiple genetic consortia to explore the association between alcohol consumption and various glaucoma-related traits, using a combination of observational, gene–environment, and MR analyses. Overall, strong and consistent adverse dose-response associations were observed for all glaucoma-related outcomes, which proved robust to a variety of sensitivity analyses. Although there was evidence for a threshold effect, specifically for inner retinal OCT measures, no quantity of alcohol consumption was found to confer a protective association with any outcome. Importantly, all adverse associations were apparent at alcohol intake below current recommended UK (112 g/week) and US (women 98 g/week; men 196 g/week) drinking guidelines.^41,42^ Additionally, the alcohol–IOP association was found to be modified by a glaucoma MTAG PRS, with the strongest associations noted in participants with the highest genetic susceptibility to glaucoma. Finally, MR experiments provided strong and consistent evidence for a causal association with mGCIPL thickness, with weaker evidence for mRNFL thickness.

Although previous studies have demonstrated adverse associations of alcohol consumption with IOP and glaucoma, results have generally been nonsignificant or inconsistent.^43–45^ A recent systematic review and meta-analysis has suggested an overall adverse association with both IOP and open-angle glaucoma, but notes that firm conclusions are limited by marked heterogeneity and a high risk of bias in the current evidence base.^7^ Importantly, most studies have not been designed specifically to explore these relationships or suffer from multiple limitations and potential biases. The evidence for inner retinal measures is more consistent, with multiple studies demonstrating adverse associations with alcohol intake.^17,46–48^

Epidemiological studies of alcohol consumption, in general, are prone to additional biases and methodological pitfalls, and no single study is ideal.^49^ However, in the absence of randomized control trials, observational studies represent the best current approach to gauging these associations. The UK Biobank, in particular, with its large sample size and wealth of glaucoma-related phenotypic and genotypic information, represents an unparalleled resource. The availability of objective structural glaucoma biomarkers, including IOP and inner retinal OCT measures, greatly increases statistical power and minimizes the risk of misclassification bias in the outcome variables. Mendelian randomization using genetic data from multiple large consortia offers an alternative approach to assessing these associations and probing causality.

To the best of our knowledge, our study is the first to simultaneously assess the association of alcohol with multiple glaucoma-related outcomes in the same cohort and the largest of the alcohol–IOP association.^7^ It is also the first to perform MR experiments and to assess whether these relationships are modified by background genetic risk of glaucoma.

Notably, we found strong dose-dependent adverse associations across all outcomes. These relationships remained significant even after adjustment for multiple potential confounding variables and proved robust to a variety of sensitivity analyses. Although causality cannot be definitively inferred, these results are supportive of a true underlying association rather than a case of residual confounding or reverse causality.

In contrast to previous studies that have suggested that adverse associations with IOP may be restricted to men, we found no differential effect or evidence of sex interaction for any outcome.^50,51^ This previously reported finding may be because of a relatively lower proportion of female drinkers in non-European populations.^2^ Despite evidence for the neuroprotective properties of polyphenols, a group of anti-inflammatory and antioxidant compounds found in high concentrations in red wine, we found no evidence for a differential or protective effect of any alcoholic beverage.^52^ This is consistent with previous studies and may be explained by the detrimental effects of alcohol on glaucoma outweighing any potential beneficial properties.^53,54^

Although the reported effect estimates for the glaucoma-related traits may seem small, it is useful to contextualize these findings. It is important to emphasize that we are comparing *between* participants, rather than *within* participants, and this always reduces effect sizes due to variability from other differences among individuals. For example, systemic β-blockers are known to have a profound IOP-lowering effect within individuals (which led to the development of topical β-blockers, a mainstay of glaucoma management), yet the difference in IOP between users and nonusers of systemic β-blockers in the UK Biobank was only 0.54 mmHg, which is similar to other population-based studies.^28,55^ Therefore, the 0.27-mmHg difference between the top and bottom quintile of alcohol consumption (even excluding nondrinkers) is considerable and suggests potentially highly clinically significant effects of alcohol within individuals. Similarly, on a population level, the effect estimates for mRNFL and mGCIPL thickness are equivalent to the average difference seen between participants separated by 10 and 5 years of age, respectively.^17^

Despite predominantly detrimental health associations, alcohol exhibits a J-shaped relationship with certain cardiovascular outcomes, with a protective effect observed at low intake. This relationship is thought to be partly accentuated by the inclusion of never drinkers in analyses and various other biases.^25^ Our restricted cubic spline regression analyses provided evidence for a threshold effect on inner retinal OCT measures, but no quantity of alcohol intake was found to be protective for any glaucoma-related outcome in this study. There was a suggestion of a threshold effect on glaucoma when including all participants, but this disappeared when restricting analyses to regular drinkers only, highlighting this potential epidemiological artifact.

There are numerous plausible biological mechanisms underlying the observed associations between alcohol and glaucoma-related traits. Chronic alcohol use is associated with various biochemical and physiological derangements, as well as a host of neurodegenerative, cardiovascular and endocrine disorders, and it is possible that the associations represent a combination of causative factors rather than a single mechanism.^2,56,57^ Alternatively, glaucoma-related outcome measures may be influenced by different underlying pathways, and this may account for the observed difference in the modeled associations between alcohol with IOP or glaucoma (log–linear effect), and mRNFL thickness or mGCIPL thickness (threshold effect) in this study.

It is well established that alcohol has neurotoxic properties, with habitual consumption associated with decreased brain volumes, peripheral neuropathy, and neurodegenerative disorders including Alzheimer’s and Parkinson’s diseases.^6,58,59^ Because the retina represents an extension of the central nervous system, with known associations of retinal layer thickness and brain volumes, this may constitute a major etiological factor.^60^ Proposed underlying mechanisms for these associations include: oxidative stress leading to free radical damage to nerves, activation of the sympatho-adrenal and hypothalamo-pituitary-adrenal axes, nutritional deficiencies (especially thiamine), and direct toxic and proinflammatory effects.^59^ Indeed, the adverse alcohol–inner retinal thickness association is the most consistent in the current literature, and our MR experiments provided strong and consistent evidence for a causal association, especially with mGCIPL thickness.

Similarly, oxidative stress-mediated damage to the trabecular meshwork may account for the observed alcohol–IOP association, which may further contribute to glaucoma risk through traditional IOP-dependent mechanisms. Our gene–environment interaction analyses showed that this association was stronger in individuals with a higher genetic risk of glaucoma. A similar interaction has been demonstrated for caffeine intake,^27^ suggesting the hypothesis that these dietary associations may reflect a combination of environmental exposure and genetically determined functional reserve in the aqueous outflow pathways.

Additionally, the observed associations may be related to the detrimental cardiovascular effects of heavy drinking, including hypertension and atherosclerosis, which may have implications for glaucomatous neurodegeneration through IOP-independent mechanisms.^61,62^ Although all associations were noted to attenuate after adjustment for systolic blood pressure in our analyses, this did not account for a significant difference in the overall results, and results were materially unchanged when restricting analyses to participants without hypertension.

It is important to acknowledge several limitations of our study. The UK Biobank response rate was only 5.5% and it has been reported that participants drank less alcohol and had lower rates of disease than the general population.^63^ Despite this “healthy volunteer” selection bias, the fact that an alcohol–glaucoma association was observed may imply that the true association in the general population is even stronger and does not negate the internal validity of our findings. Exposure ascertainment through self-reported alcohol consumption from a single questionnaire is subject to both recall and social desirability bias and may lead to significant misclassification. Furthermore, this measure may not accurately reflect alcohol consumption over the life course or specific drinking patterns. However, our alcohol intake measure did demonstrate expected associations with known alcohol-related biochemical parameters, including high-density lipoprotein cholesterol and mean corpuscular volume, providing a measure of construct validity. The presence of systemic misclassification bias (i.e., underreporting) would also not necessarily negate any observed associations, although it may have implications for quantifying threshold effects or degrees of risk and may have contributed to our finding that a higher risk was observed at alcohol intake below current recommended drinking guidelines. The cross-sectional study design evaluated all outcomes at a single timepoint, which limits our ability to make causal inferences. Although the MR analyses provided an alternative assessment of dose-response associations, life-course exposures, and causal relationships, these results were not consistent across all glaucoma-related outcomes. These analyses may also be influenced by violations of the IV assumptions, particularly horizontal pleiotropy. For example, our alcohol intake IV may be more reflective of an underlying genetic propensity to addiction, potentially implicating multiple alternative pathways and accounting for the discrepancy. Our definition of glaucoma was not specific and relied largely on participant self-report, which may again result in biases related to outcome misclassification. Finally, our results may not be generalizable to other populations and ethnic groups, as the vast majority of our study cohort were of European descent, although this does not necessarily impact the internal validity of our findings.

In conclusion, our study implicates alcohol consumption as a potentially modifiable risk factor for glaucoma, with adverse associations noted at quantities below current UK and US drinking guideline recommendations. Although it would be important for these results to be replicated in independent cohorts and ethnically diverse populations, in the absence of viable alternative study designs, our findings may be of particular interest to people with or at risk of glaucoma and their advising physicians. The presence of an underlying causal association may have important clinical and public implications and may lead to targeted lifestyle recommendations for glaucoma. This study also adds to the growing body of literature implicating gene–environment interactions in glaucoma,^27^ raising the possibility of precision nutrition and dietary recommendations based on genomic data in the future.^64^ This may be of particular importance as a preventative strategy in healthy individuals identified to be at high genetic risk of glaucoma but before the development of disease.

## Supplementary Material

Members of consortium

Suppl Fig S1

Appendix

Suppl Fig S3

Suppl Table S5

Suppl Fig S5

Suppl Table S1

Suppl Table S4

Suppl Table S3

Suppl Table S8

Suppl Table S10

Suppl Table S9

Suppl Table S11

Suppl Table S7

Suppl Table S12

## Figures and Tables

**Table 2.** Participant Characteristics by Cohort

	Analysis of IOP	Analysis of OCT Parameters	Analysis of Glaucoma Status

Sample size	81 324	36 143	84 655
Age (years), mean (SD)	56.6 (8.1)	56.3 (8.1)	56.6 (8.1)
Sex, n (%)			
Women	43 214 (53.1)	18 835 (52.1)	44 970 (53.1)
Men	38 110 (46.9)	17 308 (47.9)	39 685 (46.9)
Ethnicity, n (%)			
White	73 548 (90.4)	33 081 (91.5)	76 677 (90.6)
Black	2642 (3.3)	1071 (3.0)	2720 (3.2)
Other	5134 (6.3)	1991 (5.5)	5258 (6.2)
Townsend deprivation index, mean (SD)	−1.1 (2.9)	−1.1 (2.9)	−1.1 (2.9)
Body mass index (kg/m^2^), mean (SD)	27.3 (4.7)	27.2 (4.7)	27.3 (4.7)
Height (cm), mean (SD)	168.9 (9.3)	169.3 (9.2)	168.9 (9.3)
Systolic blood pressure (mmHg), mean (SD)	137.0 (18.3)	136.8 (18.3)	137.1 (18.3)
Spherical equivalent (D), mean (SD)	−0.4 (2.7)	0.0 (2.0)	−0.4 (2.7)
Diabetes, n (%)	4411 (5.4)	1782 (4.9)	4616 (5.5)
Smoking status, n (%)			
Never	46 741 (57.5)	20 542 (56.8)	48 652 (57.5)
Previous	29 248 (36.0)	13 280 (36.7)	30 458 (36.0)
Current	5335 (6.6)	2321 (6.4)	5545 (6.6)
Smoking intensity (cigarettes/day), mean (SD)			
Current smokers	14.5 (8.2)	13.8 (7.8)	14.5 (8.3)
Physical activity (MET-minutes/week), mean (SD)	2669 (2 678)	2692 (2 706)	2666 (2 676)
Intraocular pressure (mmHg), mean (SD)	16.1 (3.4)	—	—
mRNFL thickness (mm), mean (SD)	—	28.9 (3.8)	—
mGCIPL thickness (mm), mean (SD)	—	75.2 (5.2)	—
Glaucoma, n (%)	—	—	1 493 (1.8)
Alcohol consumption frequency, n (%)			
Never	3 906 (4.8)	1 536 (4.3)	4 077 (4.8)
Infrequent	9 700 (11.9)	4 184 (11.6)	10 097 (11.9)
Regular	64 803 (79.7)	29 136 (80.6)	67 421 (79.6)
Former	2915 (3.6)	1287 (3.6)	3060 (3.6)
Alcohol intake quantity (g/week), median (IQR)			
Infrequent	2.8 (0.0–7.9)	2.8 (0.0–8.1)	2.8 (0.0–7.9)
Regular	91.3 (43.3–170.9)	92.8 (44.6–173.5)	91.8 (43.8–171.6)
Glaucoma MTAG, mean (SD)^1^	−0.04 (1.04)	−0.05 (1.04)	−0.04 (1.04)

*Note:* n = 50 455 (IOP), n = 22 697 (OCT), n = 52 481 (glaucoma).

D — diopter. IOP — intraocular pressure; IQR — interquartile range; MET — metabolic equivalent of task; mGCIPL — macular ganglion cell—inner plexiform layer; mRNFL — macular retinal nerve fiber layer; MTAG — multitrait analysis of GWAS (genome-wide association study); SD — standard deviation.

**Table 6.** Association of Alcohol Consumption Frequency and Alcohol Intake Quantity with Intraocular Pressure, Inner Retinal OCT Measures and Glaucoma

	IOP (mmHg)			mRNFL (μm)			mGCIPL (μpm)			Glaucoma (%)		
	*β*	*95% CI*	P	*β*	*95% CI*	P	*β*	*95% CI*	P	OR	*95% CI*	P

**Alcohol consumption frequency**												
Never	0.09	(−0.04, 0.21)	0.17	−0.08	(−0.31, 0.14)	0.46	−0.08	(−0.38, 0.21)	0.57	1.23	(0.94, 1.62)	0.13
Infrequent	Reference			Reference			Reference			Reference		
Regular	**0.17**	**(0.10, 0.24)**	**<0.001**	−0.10	(−0.23, 0.02)	0.11	**−0.17**	**(−0.33, 0.00)**	**0.049**	1.13	(0.95, 1.34)	0.16
Former	**−0.15**	**(−0.28, -0.01)**	**0.03**	−0.21	(−0.45, 0.02)	0.08	−0.06	(−0.37, 0.25)	0.69	**1.53**	**(1.16, 2.02)**	**0.002**
**Alcohol intake quantity (g/week)**												
Per SD increase	**0.08**	**(0.05, 0.11)**	**<0.001**	**−0.17**	**(−0.22, −0.12)**	**<0.001**	**−0.34**	**(−0.40, −0.27)**	**<0.001**	**1.11**	**(1.05, 1.18)**	**<0.001**
Quintiles												
Quintile 1	Reference			Reference			Reference			Reference		
Quintile 2	**0.09**	**(0.01, 0.17)**	**0.02**	0.01	(−0.13, 0.14)	0.91	0.04	(−0.14, 0.22)	0.65	1.07	(0.88, 1.31)	0.48
Quintile 3	**0.15**	**(0.07, 0.23)**	**<0.001**	−0.12	(−0.26, 0.02)	0.09	−0.18	(−0.36, 0.01)	0.06	1.10	(0.90, 1.34)	0.37
Quintile 4	**0.18**	**(0.09, 0.26)**	**<0.001**	**−0.25**	**(−0.39, −0.11)**	**<0.001**	**−0.34**	**(−0.53, −0.15)**	**<0.001**	**1.22**	**(1.00, 1.48)**	**0.05**
Quintile 5	**0.27**	**(0.19, 0.36)**	**<0.001**	**−0.41**	**(−0.56, −0.27)**	**<0.001**	**−0.83**	**(−1.02, −0.63)**	**<0.001**	**1.36**	**(1.12, 1.66)**	**0.002**
*P*_trend_			**<0.001**			**<0.001**			**<0.001**			**0.001**

*Note:* Alcohol intake quantified in regular drinkers only. Details of alcohol intake quintiles for each cohort are reported in Table S3. All models adjusted for age (years), sex (women, men), ethnicity (White, Black, Other), Townsend deprivation index, assessment season (Summer, Autumn, Winter, Spring), body mass index (kg/m^2^), height (cm), systolic blood pressure (mmHg), spherical equivalent (diopters), diabetes (yes, no), smoking status (never, previous, current), smoking intensity (number of cigarettes smoked/day), physical activity (MET-minutes/week). One standard deviation increase in alcohol intake is equivalent to an additional 111 — 112 g/week.

β = beta coefficient; CI = confidence interval; IOP = intraocular pressure; MET = metabolic equivalents; mGCIPL = macular ganglion cell—inner plexiform layer; mRNFL = macular retinal nerve fiber layer; OR = odds ratio; SD = standard deviation.

**Table 13.** Results of MR analyses for alcohol intake on glaucoma-related traits

MR method	IOP (mrnHg)		mRNFL thickness (μm)		mGCIPL thickness (μm)		vCDR		POAG (log odds)	
	*Estimate (95% CI)*	P	*Estimate (95% CI)*	P	*Estimate (95% CI)*	P	*Estimate (95% CI)*	P	*Estimate (95% CI)*	P

**Full instrument (including rs 1229984)**										
IVW	−0.21 (−0.69, 0.28)	0.40	−0.63 (−1.43, 0.18)	0.13	**−1.52 (−2.55, −0.50)**	**0.004**	0.02 (−0.01, 0.04)	0.22	−0.17 (−0.51, 0.16)	0.32
Weighted median	−0.21 (−0.70, 0.29)	0.41	0.11 (−0.96, 1.19)	0.84	−0.59 (−1.99, 0.80)	0.41	0.00 (−0.05, 0.04)	0.97	−0.21 (−0.65, 0.23)	0.36
Weighted mode	−0.06 (−0.57, 0.45)	0.82	0.11 (−1.09, 1.31)	0.86	−0.29 (−2.10, 1.53)	0.76	0.01 (−0.04, 0.06)	0.69	−0.20 (−0.59, 0.19)	0.31
MR-Egger	0.05 (−0.86, 0.96)	0.92	0.17 (−1.34, 1.67)	0.83	0.02 (−1.86, 1.91)	0.98	0.01 (−0.04, 0.05)	0.73	−0.07 (−0.67, 0.53)	0.82
MR-PRESSO	−0.32 (−0.73, 0.09)	0.13	N/A	–	**−1.53 (−2.45, −0.60)**	**0.002**	N/A	–	−0.16 (−0.42, 0.11)	0.26
Multivariable MR	0.05 (−0.27, 0.37)	0.77	−0.37 (−1.06, 0.33)	0.30	−1.05 (−2.00, −0.10)	0.03	0.02 (0.00, 0.04)	0.08	−0.20 (−0.46, 0.07)	0.14
**Restricted instrument (excluding rs 1229984)**										
IVW	−0.21 (−0.76, 0.35)	0.47	**−0.98 (−1.89, −0.07)**	**0.04**	**−2.07 (−3.22, −0.93)**	**<0.001**	0.02 (−0.01, 0.06)	0.13	−0.16 (−0.56, 0.24)	0.43
Weighted median	−0.16 (−0.70, 0.38)	0.56	−1.19 (−2.44, 0.06)	0.06	**−2.45 (−4.09, −0.82)**	**0.003**	0.04 (−0.01, 0.09)	0.10	−0.25 (−0.74, 0.24)	0.32
Weighted mode	0.51 (−0.34, 1.36)	0.25	−1.37 (−3.32, 0.58)	0.17	−2.66 (−5.48, 0.16)	0.07	0.08 (0.00, 0.15)	0.06	−0.23 (−0.97, 0.52)	0.55
MR-Egger	0.62 (−1.04, 2.26)	0.46	−0.93 (−3.54, 1.67)	0.48	−1.14 (−4.44, 2.13)	0.50	0.05 (−0.05, 0.14)	0.31	0.30 (−0.88, 1.49)	0.62
MR-PRESSO	−0.30 (−0.77, 0.16)	0.20	N/A	–	**−2.09 (−3.11, −1.06)**	**<0.001**	N/A	–	−0.14 (−0.46, 0.18)	0.40
Multivariable MR	0.12 (−0.25, 0.49)	0.51	−0.65 (−1.44, 0.15)	0.11	**−1.51 (−2.61, −0.41)**	**0.007**	**0.03 (0.00, 0.06)**	**0.03**	−0.22 (−0.53, 0.18)	0.18

*Note:* No estimate is generated under the MR-PRESSO method if significant outliers are not detected. Multivariable MR adjusted for genetically determined smoking initiation.

CI = confidence interval; IOP = infraocular pressure; IVW = inverse-variance weighted; mGCIPL = macular ganglion cell—inner plexiform layer; MR = Mendelian randomization; MR-PRESSO = Mendelian Randomization-Pleiotropy Residual Sum and Outlier; mRNFL = macular retinal nerve fiber layer; N/A = not applicable; POAG = primary open-angle glaucoma; vCDR = vertical cup-to-disc ratio.

## Abbreviations and Acronyms:

CI: confidence interval
GWAS: genome-wide association study
ICD: International Classification of Diseases
IOP: intraocular pressure
IV: instrumental variable
IVN: inverse variant weighted
mGCIPL: macular ganglion cell–inner plexiform layer
MR: Mendelian randomization
mRNFL: macular retinal nerve fiber layer
MTAG: multitrait analysis of GWAS
PRS: polygenic risk score
SD: standard deviation
SNP: single nucleotide polymorphism
vCDR: vertical cup disc ratio
