# Supplementary material for: The Association of Alcohol Consumption with Glaucoma and Related Traits: Findings from the UK Biobank
Source: Ophthalmol Glaucoma. Author manuscript; Available in PMC 2023 Aug 21. (PMC10239785; doi:10.1016/j.ogla.2022.11.008)
Supplement: Members of consortium [file NIHMS1876579-supplement-Members_of_consortium.pdf]

Members of the Modifiable Risk Factors for Glaucoma Collaboration

Hugues Aschard, Institute Pasteur

Mark Chia, UCL Institute of Ophthalmology

Sharon Chua, UCL Institute of Ophthalmology

Ron Do, Icahn School of Medicine at Mount Sinai

Paul Foster, UCL Institute of Ophthalmology

Jae Kang, Brigham and Women's Hospital, Harvard Medical School

Alan Kastner, Moorfields Eye Hospital

Anthony Khawaja, UCL Institute of Ophthalmology

Jihye Kim, Harvard T.H. Chan School of Public Health

Marleen Lentjes, Örebro University

Robert Luben, UCL Institute of Ophthalmology

Kian Madjedi, UCL Institute of Ophthalmology, University of Calgary

Giovanni Montesano, Moorfields Eye Hospital

Louis Pasquale, Icahn School of Medicine at Mount Sinai

Kelsey Stuart, UCL Institute of Ophthalmology

Alasdair Warwick, UCL Institute of Cardiovascular Science

Janey Wiggs, Massachusetts Eye and Ear Infirmary, Harvard Medical School

Members of the UK Biobank Eye and Vision Consortium

Naomi Allen, University of Oxford

Tariq Aslam, The University of Manchester

Denize Atan, University of Bristol

Sarah Barman, Kingston University

Jenny Barrett, University of Leeds

Paul Bishop, The University of Manchester

Graeme Black, The University of Manchester

Tasanee Braithwaite, St Thomas' Hospital

Roxana Carare, University of Southampton

Usha Chakravarthy, Queen's University Belfast

Michelle Chan, Moorfields Eye Hospital

Sharon Chua, UCL Institute of Ophthalmology

Alexander Day, Moorfields Eye Hospital

Parul Desai, Moorfields Eye Hospital

Bal Dhillon, University of Edinburgh

Andrew Dick, University of Bristol

Alexander Doney, University of Dundee

Cathy Egan, Moorfields Eye Hospital

Sarah Ennis, University of Southampton

Paul Foster, UCL Institute of Ophthalmology

Marcus Fruttiger, UCL Institute of Ophthalmology

John Gallacher, University of Oxford

David (Ted) Garway-Heath, UCL Institute of Ophthalmology

Jane Gibson, University of Southampton

Jeremy Guggenheim, Cardiff University

Chris Hammond, King's College London

Alison Hardcastle, UCL Institute of Ophthalmology

Simon Harding, University of Liverpool

Ruth Hogg, Queen's University Belfast

Pirro Hysi, King's College London

Pearse Keane, UCL Institute of Ophthalmology

Peng Tee Khaw, UCL Institute of Ophthalmology

Anthony Khawaja, Moorfields Eye Hospital

Gerassimos Lascaratos, Moorfields Eye Hospital

Thomas Littlejohns, University of Oxford

Andrew Lotery, University of Southampton

Phil Luthert, UCL Institute of Ophthalmology

Tom MacGillivray, University of Edinburgh

Sarah Mackie, University of Leeds

Bernadette McGuinness, Queen's University Belfast

Gareth McKay, Queen's University Belfast

Martin McKibbin, Leeds Teaching Hospitals NHS Trust

Tony Moore, UCL Institute of Ophthalmology

James Morgan, Cardiff University

Eoin O'Sullivan, King's College Hospital

Richard Oram, University of Exeter

Chris Owen, St George's, University of London

Praveen Patel, Moorfields Eye Hospital

Euan Paterson, Queen's University Belfast

Tunde Peto, Queen's University Belfast

Axel Petzold, UCL Institute of Neurology

Nikolas Pontikos, UCL Institute of Ophthalmology

Jugnoo Rahi, UCL Institute of Child Health

Alicja Rudnicka, St George's, University of London

Naveed Sattar, University of Glasgow

Jay Self, University of Southampton

Panagiotis Sergouniotis, The University of Manchester

Sobha Sivaprasad, Moorfields Eye Hospital

David Steel, Newcastle University

Irene Stratton, Gloucestershire Hospitals NHS Foundation Trust

Nicholas Strouthidis, Moorfields Eye Hospital

Cathie Sudlow, University of Edinburgh

Zihan Sun, UCL Institute of Ophthalmology

Robyn Tapp, St George's, University of London

Dhanes Thomas, Moorfields Eye Hospital

Emanuele Trucco, University of Dundee

Adnan Tufail, Moorfields Eye Hospital

Ananth Viswanathan, Moorfields Eye Hospital

Veronique Vitart, University of Edinburgh

Mike Weedon, University of Exeter

Katie Williams, King's College London

Cathy Williams, University of Bristol

Jayne Woodside, Queen's University Belfast

Max Yates, University of East Anglia

Jennifer Yip, University of Cambridge

Yalin Zheng, University of Liverpool

Members of the International Glaucoma Genetics Consortium

Tin Aung, Singapore National Eye Centre

Kathryn Burdon, University of Tasmania

Li Chen, The Chinese University of Hong Kong

Ching-Yu Cheng, National University of Singapore

Jamie Craig, Flinders University

Angela Cree, University of Southampton

Victor de Vries, Erasmus Medical Centre

Sjoerd Driessen, Erasmus Medical Centre

John Fingert, University of Iowa

Paul Foster, UCL Institute of Ophthalmology

Puya Gharahkhani, QIMR Berghofer Medical Research Institute

Christopher Hammond, King's College London

Caroline Hayward, University of Edinburgh

Alex Hewitt, University of Tasmania, University of Melbourne

Pirro Hysi, King's College London

Nomdo Jansonius, University of Groningen

Fridbert Jonansson, University of Iceland

Jost Jonas, Institute of Molecular and Clinical Ophthalmology Basel

Michael Kass, Washington University

Anthony Khawaja, UCL Institute of Ophthalmology

Chiea Khor, Genome Institute of Singapore

Caroline Klaver, Erasmus Medical Centre, Radboud University Medical Centre

Jacyline Koh, Singapore National Eye Centre

Andrew Lotery, University of Southampton

Stuart MacGregor, QIMR Berghofer Medical Research Institute

David Mackey, University of Western Australia

Paul Mitchell, University of Sydney

Calvin Pang, The Chinese University of Hong Kong

Louis Pasquale, Icahn School of Medicine at Mount Sinai

Francesca Pasutto, Friedrich-Alexander-Universität Erlangen-Nürnberg,

Norbert Pfeiffer, University Medical Centre Mainz

Ozren Polašek, University of Split

Wishal Ramdas, Erasmus Medical Centre

Alexander Schuster, University Medical Centre Mainz

Ayellet Segrè, Massachusetts Eye and Ear Infirmary, Harvard Medical School

Einer Stefansson, University of Iceland

Kári Stefánsson, deCODE genetics/Amgen Inc.

Gudmar Thorleifsson, deCODE genetics/Amgen Inc.

Unnur Thorsteinsdottir, deCODE genetics/Amgen Inc., University of Iceland

Cornelia van Duijn, University of Oxford

Joëlle Vergroesen, Erasmus Medical Centre

Ananth Viswanathan, UCL Institute of Ophthalmology

Veronique Vitart, University of Edinburgh

Eranga Vithana, Singapore National Eye Centre

Janey Wiggs, Massachusetts Eye and Ear Infirmary, Harvard Medical School

James Wilson, University of Edinburgh

Robert Wojciechowski, Johns Hopkins Bloomberg School of Public Health

Tien Wong, Singapore National Eye Centre

Terri Young, University of Wisconsin-Madison
