# Supplementary figures and images for: The Association of Alcohol Consumption with Glaucoma and Related Traits: Findings from the UK Biobank
Source: Ophthalmol Glaucoma. Author manuscript; Available in PMC 2023 Aug 21. (PMC10239785; doi:10.1016/j.ogla.2022.11.008)

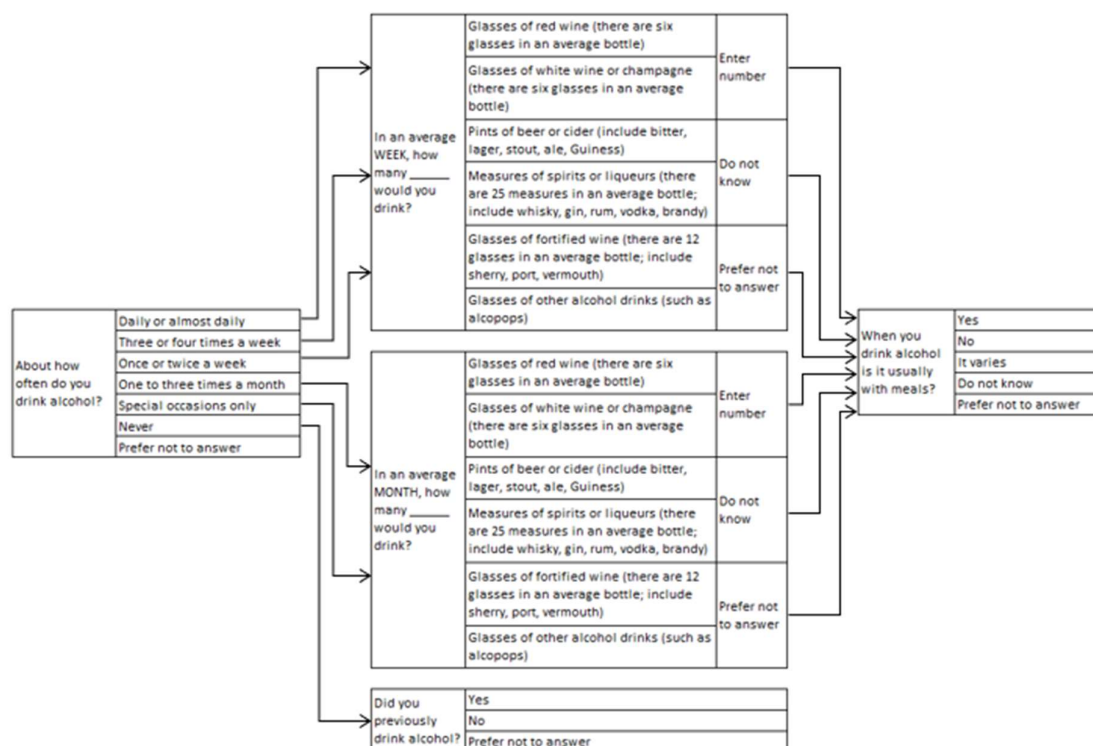

**Supplementary Figure S1.** UK Biobank baseline alcohol questionnaire flow used for this study

Supplement: Suppl Fig S1 [file NIHMS1876579-supplement-Suppl_Fig_S1.pdf]
