# Supplementary material for: The Association of Alcohol Consumption with Glaucoma and Related Traits: Findings from the UK Biobank
Source: Ophthalmol Glaucoma. Author manuscript; Available in PMC 2023 Aug 21. (PMC10239785; doi:10.1016/j.ogla.2022.11.008)
Supplement: Appendix [file NIHMS1876579-supplement-Appendix.pdf]

The association of alcohol consumption with glaucoma and related traits: findings from the UK Biobank

APPENDIX: MENDELIAN RANDOMIZATION ANALYSES

Study design

We conducted two-sample Mendelian randomization (MR) analyses, using summary-level data from published genome-wide association studies (GWAS), to test for the causal association between genetically determined alcohol intake (alcoholic drinks per week) and five glaucoma-related outcomes. MR is a technique which uses genetic variants as instrumental variables (IVs) to estimate the causal effect of an exposure on an outcome.[1] By leveraging the random allocation of alleles at conception, MR allows for estimates which are unbiased by residual confounding and reverse causation. Three assumptions need to be satisfied to ensure a valid IV: (1) the IV must be associated with the exposure; (2) the IV must not be associated with any confounder of the exposure-outcome relationship; and (3) the IV must affect the outcome only through the exposure of interest. If these assumptions are satisfied, an estimated casual effect can be calculated from the observed IV-exposure and IV-outcome associations.

Instrumental variable selection

We used results from the most recent GWAS of alcohol intake from the GWAS & Sequencing Consortium of Alcohol and Nicotine use (GSCAN) consortium to guide construction of our instrumental variables.[2] The study identified 99 conditionally independent, genome-wide significant (P<5x10^{-8}) single nucleotide polymorphisms (SNPs) in a sample of 941 280 European participants. These genetic variants explain <1% of the variance in alcohol intake with a one standard deviation (SD) increase in the genetic instrument representing one additional alcoholic drink per week.[2] Genetic principal components, population stratification and participant relatedness were adjusted for in the GWAS. At loci with multiple genome-wide significant SNPs, we excluded those with linkage disequilibrium R^2>0.1 and within 10 000 kb, retaining only the SNP with the lowest *P*-value, using the 1000 Genomes Project European reference population.[3] Palindromic SNPs with minor allele frequency (MAF)>0.42, or when allele frequencies were not reported, were excluded. Effect alleles were harmonized across exposure and outcome datasets.

The rs1229984 variant in the alcohol dehydrogenase 1B (*ADH1B*) gene region is consistently and strongly associated with lower alcohol intake in European populations.[4–6] Alcohol consumption in the

presence of this genetic variant, however, leads to rapid accumulation of toxic intermediate metabolites and it is therefore also associated with higher levels of alcohol-related tissue damage.[5] Given these biological associations and the large effect size on alcohol intake compared to other SNPs (see **Figures I–V**), inclusion of this SNP in an IV may bias MR results. We therefore considered two alcohol intake IVs in our analyses: a full instrument, comprised of all genetic variants from the GSCAN GWAS including rs1229984; and a restricted instrument, comprising the same variants but excluding rs1229984. The number of SNPs included in the full and restricted IV for each outcome are reported in **Table I** and full details of these SNPs are available in **Table II**.

Outcome data sources

We utilized publicly-available summary statistics from large GWAS for intraocular pressure (IOP),[7] macular retinal nerve fiber layer thickness (mRNFL),[8] macular ganglion cell-inner plexiform layer thickness (mGCIPL),[8] vertical cup-disc-ratio (vCDR),[9] and primary open-angle glaucoma (POAG).[10] Further details of these outcome data sources are available in the main text and in **Table I**.

Statistical analyses

The main MR analyses were performed using a multiplicative random-effects inverse-variance weighted (IVW) method.[11] This method provides precise and efficient estimates but is sensitive to invalid IVs and pleiotropy.[12] We therefore conducted a variety of sensitivity analyses using four alternative MR methods: weighted median,[13] weighted mode,[14] MR-Egger[15] and MR pleiotropy residual sum and outlier (MR-PRESSO).[16] We additionally performed multivariable MR,[17] adjusting for genetically determined smoking initiation, using 378 conditionally independent, genome-wide significant SNPs associated with smoking initiation (a binary phenotype defined as any history of regular smoking) in a sample of 1 232 091 European participants from the GSCAN GWAS.[2] These genetic variants explain 2.3% of the variance in smoking initiation with a one SD increase in genetically predicted smoking initiation corresponding to a 10% increased risk of smoking.[2] Each method makes different assumptions about the nature of pleiotropy and consistent estimates across methods strengthens causal inferences.[18] The weighted median method gives consistent estimates if the majority of IVs are valid, while the weighted mode method assumes that a plurality of IVs are valid. The MR-Egger and MR-PRESSO methods can test and correct for directional pleiotropy.

Under the IVW method, we calculated the mean F statistic as an indicator of instrument strength (a value >10 is usually considered a strong instrument).[19] We assessed for heterogeneity with the I^2 and Cochran's Q statistics in the IVW model and with Rucker's Q' statistic in MR-Egger regression. The I^2_{GX} statistic is an indicator of expected relative bias (or dilution) of the MR-Egger causal estimate.[20] In MR-

Egger regression, a significant difference of the intercept from zero is evidence for average directional horizontal pleiotropy.[15] The MR-PRESSO global test evaluates for horizontal pleiotropy, the outlier test detects specific SNP outliers, and the distortion test evaluates whether there is a significant difference in the causal estimate before and after adjusting for outliers.[16] Full results of these tests and statistics are available in **Table III**.

MR estimates are presented as unit change in the outcome per one SD increase in the genetic instrument. All analyses were performed in R version 4.1.1 (https://www.R-project.org) using the *TwoSampleMR*, *MendelianRandomization* and *MRPRESSO* packages. The study details and protocol were not pre-registered.

References

1. Davies NM, Holmes M V, Davey Smith G. Reading Mendelian randomisation studies: a guide, glossary, and checklist for clinicians. BMJ. 2018;362:k601.

2. Liu M, Jiang Y, Wedow R, Li Y, Brazel DM, Chen F, et al. Association studies of up to 1.2 million individuals yield new insights into the genetic etiology of tobacco and alcohol use. Nat Genet. 2019;51(2):237–44.

3. Auton A, Abecasis GR, Altshuler DM, Durbin RM, Abecasis GR, Bentley DR, et al. A global reference for human genetic variation. Nature. 2015;526(7571):68–74.

4. Edenberg HJ. The genetics of alcohol metabolism: role of alcohol dehydrogenase and aldehyde dehydrogenase variants. Alcohol Res Heal. 2007;30(1):5–13.

5. Polimanti R, Gelernter J. ADH1B: From alcoholism, natural selection, and cancer to the human phenome. Am J Med Genet. 2018;177(2):113–25.

6. Macgregor S, Lind PA, Bucholz KK, Hansell NK, Madden PAF, Richter MM, et al. Associations of ADH and ALDH2 gene variation with self report alcohol reactions, consumption and dependence: an integrated analysis. Hum Mol Genet. 2009;18(3):580–93.

7. Khawaja AP, Cooke Bailey JN, Wareham NJ, Scott RA, Simcoe M, Igo RP, et al. Genome-wide analyses identify 68 new loci associated with intraocular pressure and improve risk prediction for primary open-angle glaucoma. Nat Genet. 2018;50(6):778–82.

8. Currant H, Hysi P, Fitzgerald TW, Gharahkhani P, Bonnemaijer PWM, Senabouth A, et al. Genetic variation affects morphological retinal phenotypes extracted from UK Biobank optical coherence tomography images. PLoS Genet. 2021;17(5):e1009497.

9. Springelkamp H, Iglesias AI, Mishra A, Höhn R, Wojciechowski R, Khawaja AP, et al. New insights into the genetics of primary open-angle glaucoma based on meta-analyses of intraocular pressure and optic disc characteristics. Hum Mol Genet. 2017;26(2):438–53.

10. Gharahkhani P, Jorgenson E, Hysi P, Khawaja AP, Pendergrass S, Han X, et al. Genome-wide meta-analysis identifies 127 open-angle glaucoma loci with consistent effect across ancestries. Nat Commun. 2021;12(1):1258.

11. Burgess S, Butterworth A, Thompson SG. Mendelian Randomization Analysis With Multiple Genetic Variants Using Summarized Data. Genet Epidemiol. 2013;37(7):658–65.

12. Burgess S, Bowden J, Fall T, Ingelsson E, Thompson SG. Sensitivity Analyses for Robust Causal Inference from Mendelian Randomization Analyses with Multiple Genetic Variants. Epidemiology. 2017;28(1):30–42.

13. Bowden J, Davey Smith G, Haycock PC, Burgess S. Consistent Estimation in Mendelian Randomization with Some Invalid Instruments Using a Weighted Median Estimator. Genet Epidemiol. 2016;40(4):304–14.

14. Hartwig FP, Davey Smith G, Bowden J. Robust inference in summary data Mendelian randomization via the zero modal pleiotropy assumption. Int J Epidemiol. 2017;46(6):1985–98.

15. Bowden J, Davey Smith G, Burgess S. Mendelian randomization with invalid instruments: effect estimation and bias detection through Egger regression. Int J Epidemiol. 2015;44(2):512–25.

16. Verbanck M, Chen C-Y, Neale B, Do R. Detection of widespread horizontal pleiotropy in causal relationships inferred from Mendelian randomization between complex traits and diseases. Nat Genet. 2018;50(5):693–8.

17. Burgess S, Thompson SG. Multivariable Mendelian randomization: the use of pleiotropic genetic variants to estimate causal effects. Am J Epidemiol. 2015;181(4):251–60.

18. Lawlor DA, Tilling K, Davey Smith G. Triangulation in aetiological epidemiology. Int J Epidemiol. 2016;45(6):1866–86.

19. Burgess S, Thompson SG. Avoiding bias from weak instruments in Mendelian randomization studies. Int J Epidemiol. 2011;40(3):755–64.

20. Bowden J, Del Greco M F, Minelli C, Davey Smith G, Sheehan NA, Thompson JR. Assessing the suitability of summary data for two-sample Mendelian randomization analyses using MR-Egger regression: the role of the I2 statistic. Int J Epidemiol. 2016;45(6):1961–74.

Table I. Details of summary-level data used for Mendelian randomization experiments

Trait	Assessment method	Source (PMID)	Number of participants	Participant ethnicity	SNPs included in the instrumental variable (full/restricted)	Maximum potential sample overlap	Covariables adjusted for
**Exposure**							
Alcohol intake	Self-report	GSCAN (30643251)	941 280	European	–	–	Age, sex, age*sex, PCs
**Outcome**							
IOP	Tonometry	UKB, EPIC-N, IGGC (29785010)	139 555	European	75/74	11.0%	Age, sex, PCs
mRNFL	OCT	UKB (33979322)	31 434	European	87/86	3.3%	Age, sex, PCs
mGCIPL	OCT	UKB (33979322)	31 434	European	87/86	3.3%	Age, sex, PCs
vCDR	SLO, ODP	IGGC (28073927)	23 899	European	75/74	0.0%	Age, sex, PCs
POAG	Multiple	IGGC (33627673)	216 257	European	76/75	8.6%	Age, sex, PCs

Abbreviations: PMID, PubMed ID; SNP, single-nucleotide polymorphism; GSCAN, GWAS and Sequencing Consortium of Alcohol and Nicotine Use; IOP, intraocular pressure; UKB, UK Biobank; EPIC-N, European Prospective Investigation into Cancer and Nutrition-Norfolk; IGGC, International Glaucoma Genetics Consortium; mRNFL, macular retinal nerve fiber layer; OCT, optical coherence tomography; mGCIPL, macular ganglion cell-inner plexiform layer; vCDR, vertical cup-disc ratio; SLO, scanning laser ophthalmoscopy; ODP, optic disc photography; POAG, primary open-angle glaucoma; PC, principal components.

Table II. Details of SNPs included in the alcohol instrumental variables and their association with glaucoma and related traits

SNP	Chr	BP	RA	EA	EAF	Alcohol intake		IOP		mRNFL		mGCIPL		vCDR		POAG	
						Beta	SE	Beta	SE	Beta	SE	Beta	SE	Beta	SE	Beta	SE
rs705687	1	4548453	A	G	0.785	-0.011	0.002	0.004	0.016	0.017	0.044	0.079	0.057	0.001	0.002	0.011	0.017
rs58107686	1	33837334	C	A	0.328	-0.010	0.002	0.029	0.014	0.015	0.038	0.070	0.051	-0.001	0.001	0.025	0.014
rs12088813	1	66407700	A	C	0.267	-0.009	0.002	0.023	0.015	0.046	0.041	0.090	0.053	0.002	0.002	0.009	0.015
rs5024204	1	71491890	A	T	0.278	0.010	0.002	-	-	-0.126	0.041	-0.045	0.054	-	-	-	-
rs10753661	1	165119792	G	A	0.684	-0.009	0.002	-0.001	0.014	0.015	0.039	-0.010	0.051	-0.001	0.001	0.001	0.014
rs28680958	1	173848808	G	A	0.217	-0.011	0.002	0.022	0.016	-0.021	0.044	-0.017	0.058	-0.004	0.002	-0.003	0.016
rs823114	1	205719532	G	A	0.553	0.009	0.001	-0.006	0.013	0.072	0.036	-0.004	0.048	0.001	0.001	-0.003	0.013
rs77165542	2	430975	C	T	0.035	-0.026	0.004	0.066	0.039	0.104	0.100	-0.047	0.131	-0.001	0.006	0.053	0.039
rs1260326	2	27730940	T	C	0.601	0.021	0.001	0.023	0.013	-0.031	0.037	0.059	0.049	0.000	0.001	-0.005	0.013
rs2178197	2	27860551	A	G	0.569	-0.009	0.001	0.028	0.013	-0.023	0.037	-0.048	0.048	0.000	0.001	0.002	0.013
rs13383034	2	45155276	C	T	0.329	0.015	0.002	-0.037	0.014	-0.031	0.040	-0.032	0.052	-0.003	0.002	-0.023	0.014
rs13032049	2	63581507	A	G	0.283	0.010	0.002	-0.046	0.015	0.042	0.041	-0.021	0.053	0.002	0.002	-0.012	0.014
rs828867	2	74334462	G	A	0.545	0.009	0.001	-0.020	0.013	-0.004	0.037	-0.058	0.049	0.002	0.001	0.010	0.013
rs11692435	2	98275354	G	A	0.085	0.017	0.003	0.022	0.024	-0.002	0.068	-0.051	0.090	0.001	0.002	0.082	0.022
rs13024996	2	144225215	C	A	0.364	-0.011	0.002	0.036	0.013	0.040	0.037	0.004	0.049	-0.001	0.001	0.016	0.013
rs72859280	2	147956293	G	T	0.036	0.023	0.004	-0.021	0.036	-0.001	0.098	-0.007	0.128	-0.005	0.004	-0.001	0.035
rs56337305	2	225475560	T	C	0.383	-0.010	0.001	0.018	0.013	-0.051	0.038	-0.042	0.049	0.002	0.001	0.008	0.013
rs13094887	3	70968431	A	T	0.301	-0.010	0.002	-	-	-0.079	0.039	-0.039	0.052	-	-	-	-
rs62250685	3	85457240	A	G	0.614	-0.014	0.002	0.042	0.013	-0.036	0.037	0.027	0.049	-0.002	0.001	0.053	0.013
rs13066454	3	93994255	C	T	0.398	-0.009	0.001	0.013	0.013	0.067	0.037	0.043	0.049	-0.002	0.001	0.021	0.013
rs9838144	3	131576287	G	C	0.209	-0.010	0.002	-	-	0.037	0.045	-0.017	0.059	-	-	-	-
rs2011092	3	141124607	T	C	0.339	-0.009	0.002	-0.035	0.014	0.016	0.038	-0.031	0.050	-0.001	0.001	-0.012	0.014
rs60654199	3	141267295	C	A	0.063	-0.017	0.003	0.029	0.027	-0.036	0.073	-0.107	0.096	0.002	0.003	0.044	0.027
rs6787172	3	158187811	T	G	0.554	-0.008	0.001	0.004	0.013	-0.021	0.037	0.030	0.048	0.001	0.001	-0.014	0.013
rs3748034	4	3446091	G	T	0.143	-0.012	0.002	-0.009	0.019	0.028	0.052	0.039	0.068	0.001	0.002	-0.030	0.020
rs7682824	4	39406254	C	T	0.548	0.008	0.002	-	-	0.027	0.051	-0.023	0.068	-	-	0.012	0.020
rs11940694	4	39414993	A	G	0.597	0.026	0.001	0.023	0.013	-0.024	0.037	-0.068	0.049	0.003	0.001	-0.013	0.013
rs4501255	4	42151306	C	G	0.235	0.011	0.002	-	-	-0.050	0.043	-0.101	0.056	-	-	-	-
rs12499107	4	99678691	A	G	0.131	0.013	0.002	0.006	0.019	0.012	0.054	-0.016	0.071	-0.001	0.002	0.012	0.020
rs1229984	4	100239319	T	C	0.963	0.151	0.004	-0.031	0.041	0.089	0.110	0.060	0.144	0.000	0.004	-0.030	0.036
rs10028756	4	100254520	G	A	0.129	-0.019	0.002	0.004	0.019	-0.028	0.053	0.063	0.070	-0.002	0.002	-0.010	0.019
rs36052336	4	100273594	A	G	0.061	-0.018	0.003	0.032	0.027	0.079	0.075	0.049	0.099	0.001	0.003	-0.002	0.027
rs2165670	4	100286085	G	A	0.106	0.023	0.002	-0.023	0.022	-0.054	0.060	-0.147	0.079	0.000	0.002	0.026	0.021
rs79139602	4	100444363	A	T	0.021	0.060	0.005	-	-	-0.184	0.126	-0.320	0.166	-	-	-	-
rs4699791	4	101243023	G	A	0.096	0.019	0.002	-0.055	0.022	-0.062	0.062	-0.081	0.082	-0.001	0.003	0.025	0.021

rs13107325	4	103188709	C	T	0.072	-0.028	0.003	-0.043	0.025	-0.063	0.070	0.020	0.091	-0.002	0.003	-0.025	0.026
rs4690727	4	143648579	C	G	0.718	0.011	0.002	-	-	-0.019	0.041	0.063	0.054	-	-	-	-
rs10004020	4	152968372	G	A	0.720	0.009	0.002	-0.011	0.014	0.004	0.040	0.023	0.053	0.000	0.002	0.010	0.014
rs4916723	5	87854395	A	C	0.416	-0.010	0.001	-0.008	0.013	0.013	0.037	0.083	0.049	-0.001	0.001	-0.005	0.013
rs12655091	5	144412335	G	A	0.530	-0.008	0.001	-0.023	0.013	-0.013	0.036	-0.060	0.048	0.002	0.001	-0.005	0.013
rs55872084	5	155902003	G	T	0.235	0.010	0.002	0.029	0.015	-0.007	0.042	-0.028	0.056	-0.001	0.002	0.009	0.015
rs10085696	7	69783020	A	G	0.186	-0.011	0.002	0.012	0.017	0.033	0.046	0.129	0.061	-0.001	0.002	-0.017	0.017
rs6460047	7	73042443	T	C	0.208	0.012	0.002	-0.023	0.016	-0.056	0.045	-0.049	0.059	0.005	0.002	-0.019	0.016
rs10236149	7	98977515	A	G	0.123	-0.013	0.002	-0.048	0.020	-0.037	0.054	-0.171	0.072	-0.001	0.002	0.004	0.019
rs35034355	7	103840115	G	A	0.521	-0.008	0.001	-0.011	0.013	0.015	0.036	0.027	0.048	0.000	0.001	-0.008	0.013
rs6951574	7	153489744	T	C	0.458	0.013	0.001	0.004	0.014	0.011	0.037	-0.051	0.048	0.000	0.002	-0.007	0.015
rs13250583	8	20949917	C	T	0.213	-0.010	0.002	0.002	0.016	0.036	0.044	0.061	0.058	0.000	0.002	0.024	0.016
rs1217091	8	64527399	T	C	0.812	0.012	0.002	-0.026	0.016	0.014	0.046	0.009	0.060	0.001	0.002	0.005	0.017
rs55932213	9	108755622	A	G	0.736	0.009	0.002	0.005	0.015	0.016	0.042	-0.077	0.056	0.003	0.002	0.014	0.015
rs10978550	9	109345993	T	C	0.206	-0.012	0.002	-0.028	0.016	0.101	0.045	-0.003	0.059	0.003	0.002	-0.009	0.016
rs7074871	10	110507806	G	A	0.255	-0.009	0.002	0.018	0.015	0.004	0.041	0.085	0.054	0.001	0.002	0.013	0.015
rs17665139	10	125093880	C	T	0.149	-0.012	0.002	0.035	0.018	0.018	0.051	-0.027	0.066	0.000	0.002	0.038	0.018
rs7950166	11	8642218	C	T	0.637	-0.010	0.002	0.043	0.013	0.120	0.038	0.050	0.050	0.001	0.001	0.004	0.014
rs11030084	11	27643725	C	T	0.184	-0.011	0.002	0.037	0.016	0.075	0.046	0.048	0.061	0.000	0.002	-0.001	0.017
rs56030824	11	47397353	G	A	0.322	-0.012	0.002	-0.102	0.014	-0.076	0.039	0.007	0.051	0.000	0.001	-0.026	0.014
rs10750025	11	113424042	C	T	0.686	0.010	0.002	-0.015	0.014	0.042	0.039	0.007	0.051	0.001	0.002	-0.016	0.014
rs1713676	11	113660576	A	G	0.522	-0.008	0.001	0.022	0.013	0.071	0.036	0.114	0.048	0.002	0.001	0.005	0.013
rs4938230	11	116075001	C	A	0.842	0.013	0.002	0.000	0.018	-0.079	0.051	-0.027	0.066	0.001	0.002	0.012	0.018
rs682011	11	121544285	T	C	0.559	0.008	0.001	0.026	0.013	-0.080	0.037	-0.180	0.048	-0.002	0.001	-0.018	0.013
rs12795042	11	133658168	A	C	0.623	-0.008	0.002	-0.006	0.014	0.046	0.038	0.073	0.050	0.000	0.002	0.003	0.014
rs10876188	12	51895882	C	T	0.457	-0.008	0.001	-0.021	0.013	-0.003	0.036	-0.008	0.048	-0.001	0.001	0.000	0.013
rs3809162	12	54674235	A	G	0.397	0.009	0.001	0.010	0.013	0.017	0.037	-0.060	0.049	0.002	0.001	-0.012	0.013
rs10506274	12	81601464	G	T	0.484	-0.009	0.001	-0.003	0.013	-0.009	0.036	0.011	0.048	-0.002	0.001	-0.003	0.013
rs4842786	12	92170791	G	A	0.584	-0.009	0.001	-0.011	0.013	0.026	0.037	-0.034	0.048	-0.001	0.001	0.009	0.014
rs500321	13	27124360	A	T	0.736	-0.010	0.002	-	-	-0.044	0.041	0.044	0.054	-	-	-	-
rs1123285	14	57274519	C	G	0.335	-0.009	0.002	-	-	0.062	0.039	0.025	0.051	-	-	-	-
rs2180870	14	58782779	T	C	0.135	-0.012	0.002	0.044	0.019	0.049	0.054	0.082	0.071	-0.001	0.002	0.011	0.019
rs28929474	14	94844947	C	T	0.018	-0.037	0.005	-0.126	0.047	0.000	0.129	-0.197	0.169	0.009	0.006	0.031	0.048
rs11625650	14	104610138	G	A	0.233	-0.010	0.002	0.006	0.016	-0.107	0.043	0.112	0.056	-0.001	0.002	0.013	0.017
rs2472297	15	75027880	C	T	0.249	0.011	0.002	0.051	0.015	0.065	0.041	0.074	0.054	0.001	0.002	0.052	0.015
rs12907323	15	86796012	A	G	0.411	0.008	0.001	0.005	0.013	0.102	0.037	0.159	0.049	0.001	0.001	0.004	0.013
rs2764771	16	20013793	G	A	0.307	0.010	0.002	0.016	0.014	0.019	0.040	0.020	0.052	-0.001	0.001	0.015	0.014
rs17177078	16	24810681	C	T	0.063	-0.022	0.003	0.003	0.027	-0.123	0.078	-0.092	0.102	-0.001	0.003	-0.049	0.027
rs378421	16	28754684	G	A	0.404	-0.011	0.001	-0.039	0.015	0.057	0.037	0.018	0.048	-0.003	0.003	-0.030	0.014

rs113443718	16	29892184	G	A	0.305	-0.010	0.002	-0.002	0.015	0.002	0.039	0.003	0.052	0.000	0.002	0.030	0.015
rs62044525	16	64872590	C	G	0.184	-0.012	0.002	-	-	0.088	0.047	0.070	0.061	-	-	-	-
rs7185555	16	69131281	G	C	0.153	-0.011	0.002	-	-	0.015	0.050	0.098	0.065	-	-	-	-
rs79616692	16	72338507	G	C	0.108	0.016	0.002	-	-	-0.015	0.058	-0.089	0.077	-	-	-	-
rs4548913	17	2209888	G	A	0.632	-0.008	0.002	0.054	0.014	-0.004	0.038	0.002	0.050	0.001	0.002	0.055	0.014
rs3803800	17	7462969	A	G	0.786	0.011	0.002	-0.013	0.016	0.040	0.044	0.098	0.058	0.000	0.002	0.000	0.016
rs2854334	17	29715500	A	G	0.615	0.009	0.001	-0.047	0.013	-0.022	0.037	-0.033	0.049	0.001	0.001	-0.032	0.013
rs10438820	17	78524597	C	T	0.702	0.009	0.002	-0.003	0.014	-0.069	0.039	-0.084	0.052	0.000	0.001	0.010	0.014
rs9950000	18	53052169	C	T	0.395	-0.009	0.001	-0.016	0.013	-0.028	0.037	0.034	0.049	0.000	0.001	-0.031	0.013
rs4092465	18	55080437	A	G	0.635	-0.008	0.002	-0.017	0.014	-0.004	0.038	-0.027	0.050	0.001	0.001	0.006	0.014
rs281379	19	49214274	G	A	0.508	0.014	0.001	-0.008	0.013	-0.041	0.036	-0.121	0.048	0.002	0.001	0.004	0.013
rs4815364	20	25035711	G	A	0.616	0.009	0.001	-0.015	0.013	-0.052	0.037	-0.001	0.049	0.000	0.001	-0.019	0.013
rs9607814	22	41946519	C	A	0.200	-0.010	0.002	0.025	0.016	0.121	0.046	0.133	0.060	-0.001	0.002	0.015	0.016

Abbreviations: SNP, single-nucleotide polymorphism; Chr, chromosome; BP, base position (build 37); RA, reference allele; EA, effect allele; EAF, effect allele frequency; IOP, intraocular pressure; mRNFL, macular retinal nerve fiber layer; mGCIPL, macular ganglion cell-inner plexiform layer; vCDR, vertical cup-disc ratio; POAG, primary open-angle glaucoma; SE, standard error.

Notes: rs1229984 (highlighted in green) located in the alcohol dehydrogenase 1B (*ADH1B*) gene region is the SNP with the single largest effect on alcohol intake. This SNP was excluded from the restricted alcohol instrumental variable (see text for details).

Table III. Tests of heterogeneity, directional pleiotropy and regression dilution statistics for the alcohol instrumental variables

	**IOP**		**mRNFL**		**mGCIPL**		**vCDR**		**POAG**	
	Estimate	*P*-value	Estimate	*P*-value	Estimate	*P* -value	Estimate	*P* -value	Estimate	*P* -value
**Full instrument (including rs1229984)**										
**IVW**										
Mean *F* statistic	67.0	-	64.5	-	64.5	-	67.0	-	67.4	-
Cochran's *Q* statistic	259.6 (74)	<0.0001	123.0 (86)	0.005	114.8 (86)	0.02	74.8 (74)	0.45	135.5 (75)	<0.0001
I^2 statistic	71.5%	-	30.1%	-	25.1%	-	1.0%	-	44.6%	-
**MR-Egger**										
Rucker's *Q'* statistic	258.1 (73)	<0.0001	120.9 (85)	0.006	110.1 (85)	0.04	74.6 (73)	0.43	135.2 (74)	<0.0001
I^2_{GX} statistic	93.9%	-	93.3%	-	93.3%	-	94.5%	-	94.2%	-
Intercept	0.00	0.52	-0.01	0.22	-0.02	0.06	0.00	0.67	0.00	0.69
**MR-PRESSO**										
Global test	-	<0.0001	-	0.005	-	0.02	-	0.45	-	<0.0001
Number of outliers	3	-	0	-	2	-	0	-	4	-
Distortion test	36.1%	0.67	N/A	-	29.7%	0.99	N/A	-	-10.6%	0.87
**Restricted instrument (excluding rs1229984)**										
**IVW**										
Mean *F* statistic	47.6	-	47.5	-	47.5	-	47.6	-	47.2	-
Cochran's *Q* statistic	259.6 (73)	<0.0001	119.4 (85)	0.008	109.6 (85)	0.04	73.9 (73)	0.42	135.5 (74)	<0.0001
I^2 statistic	71.9%	-	28.8%	-	22.4%	-	1.2%	-	45.4%	-
**MR-Egger**										
Rucker's *Q'* statistic	255.8 (72)	<0.0001	119.4 (84)	0.007	109.1 (84)	0.04	73.6 (72)	0.42	134.3 (73)	<0.0001
I^2_{GX} statistic	71.8%	-	72.3%	-	72.3%	-	76.6%	-	74.1%	-
Intercept	-0.01	0.30	0.00	0.97	-0.01	0.55	0.00	0.59	-0.01	0.42
**MR-PRESSO**										
Global test	-	<0.0001	-	0.009	-	0.04	-	0.44	-	<0.0001
Number of outliers	4	-	0	-	2	-	0	-	4	-
Distortion test	32.6%	0.77	N/A	-	0.7%	0.98	N/A	-	-17.3%	0.86

Abbreviations: IOP, intraocular pressure; mRNFL, macular retinal nerve fiber layer; mGCIPL, macular ganglion cell-inner plexiform layer; vCDR, vertical cup-disc ratio; POAG, primary open-angle glaucoma; CI, confidence interval; IVW, inverse variance weighted; MR-Egger, Mendelian Randomization-Egger; MR-PRESSO, Mendelian Randomization-Pleiotropy Residual Sum and Outlier; N/A, not applicable.

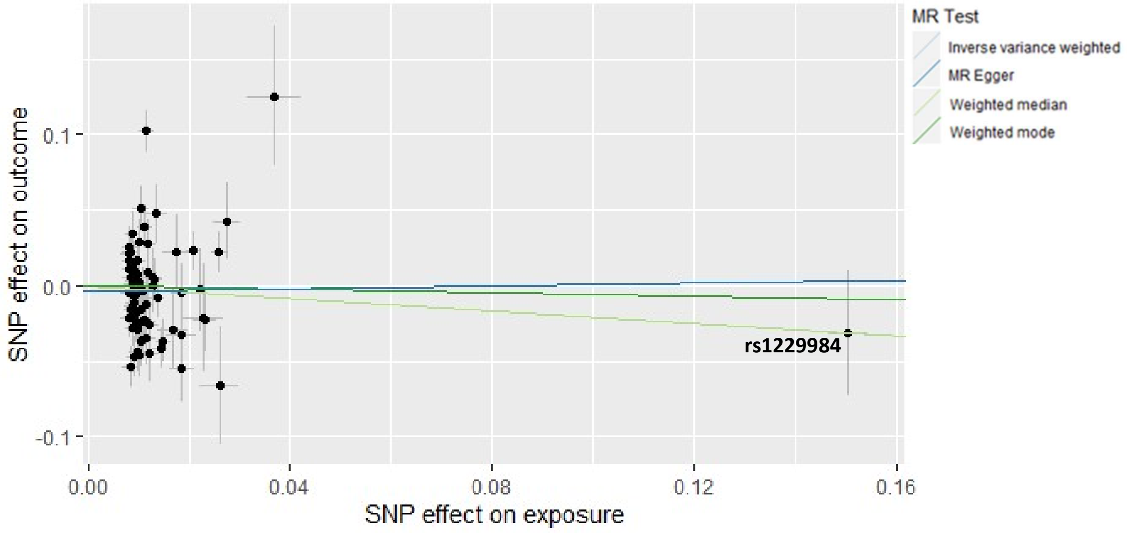

Figure I. Scatter plot of the SNP-alcohol intake (exposure) and SNP-IOP (outcome) association estimates for the full alcohol instrument

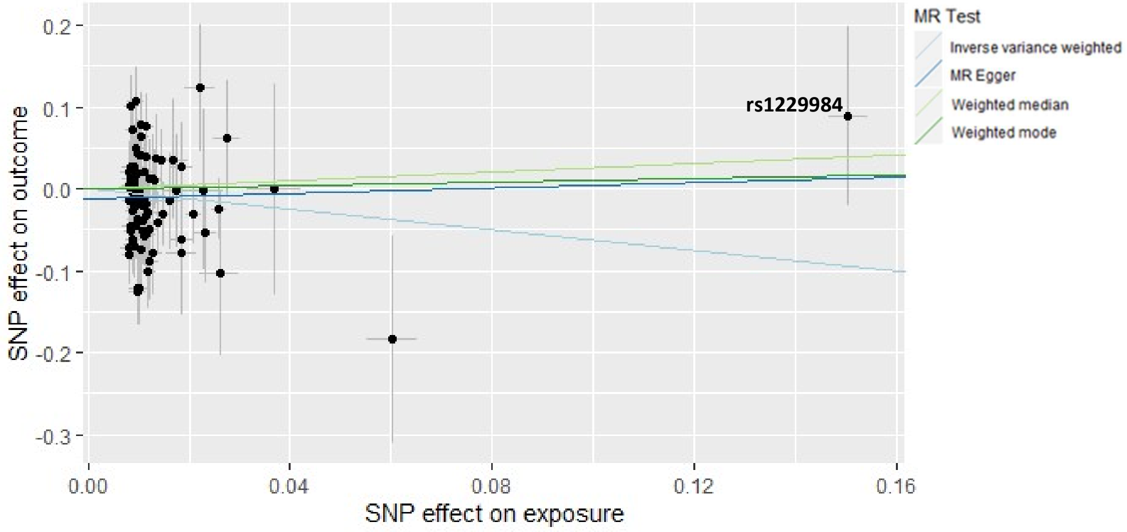

Figure II. Scatter plot of the SNP-alcohol intake (exposure) and SNP-mRNFL (outcome) association estimates for the full alcohol instrument

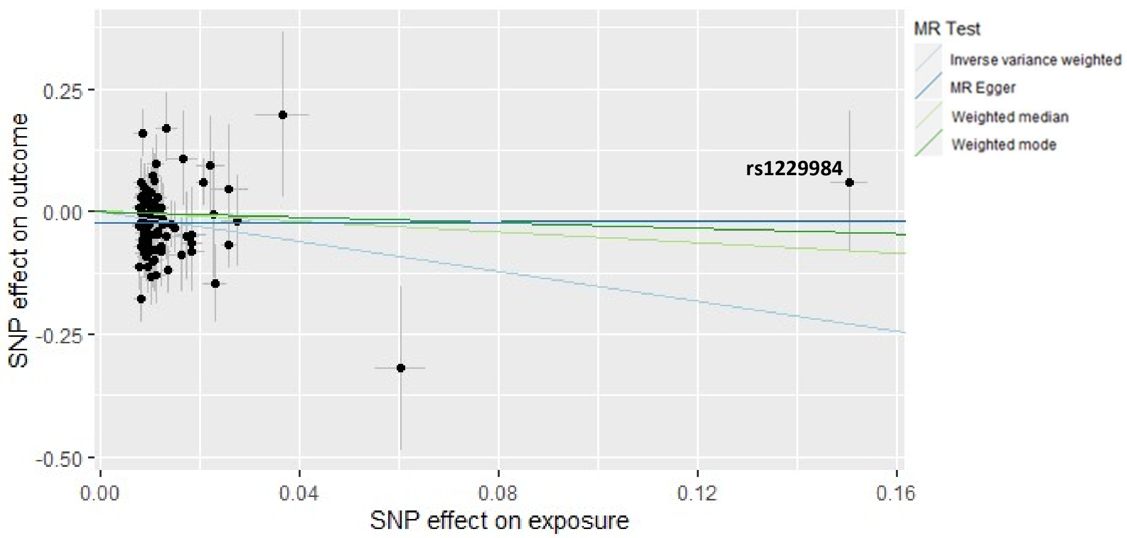

Figure III. Scatter plot of the SNP-alcohol intake (exposure) and SNP-mGCIPL (outcome) association estimates for the full alcohol instrument

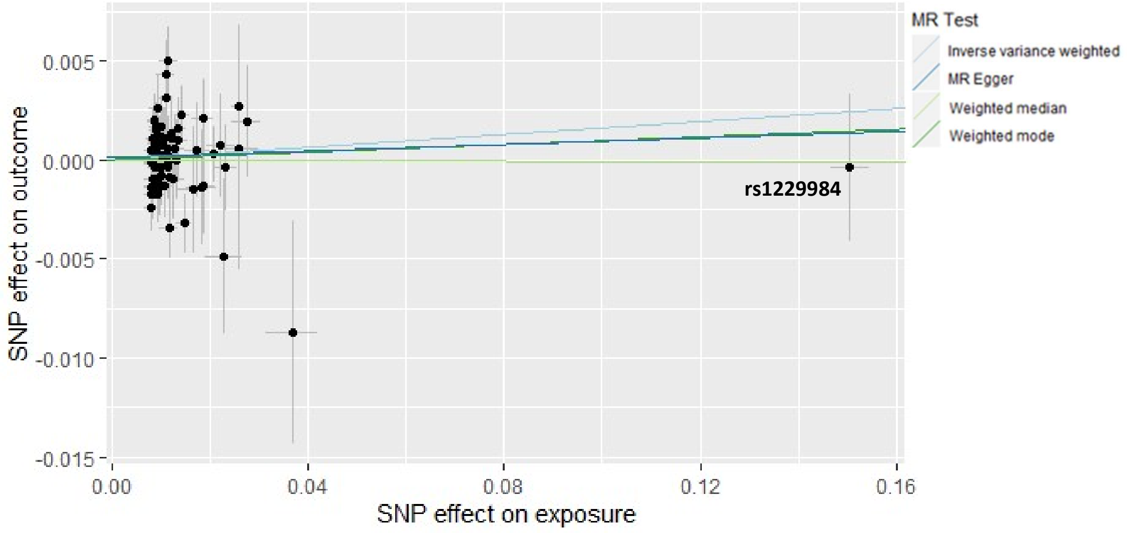

Figure IV. Scatter plot of the SNP-alcohol intake (exposure) and SNP-vCDR (outcome) association estimates for the full alcohol instrument

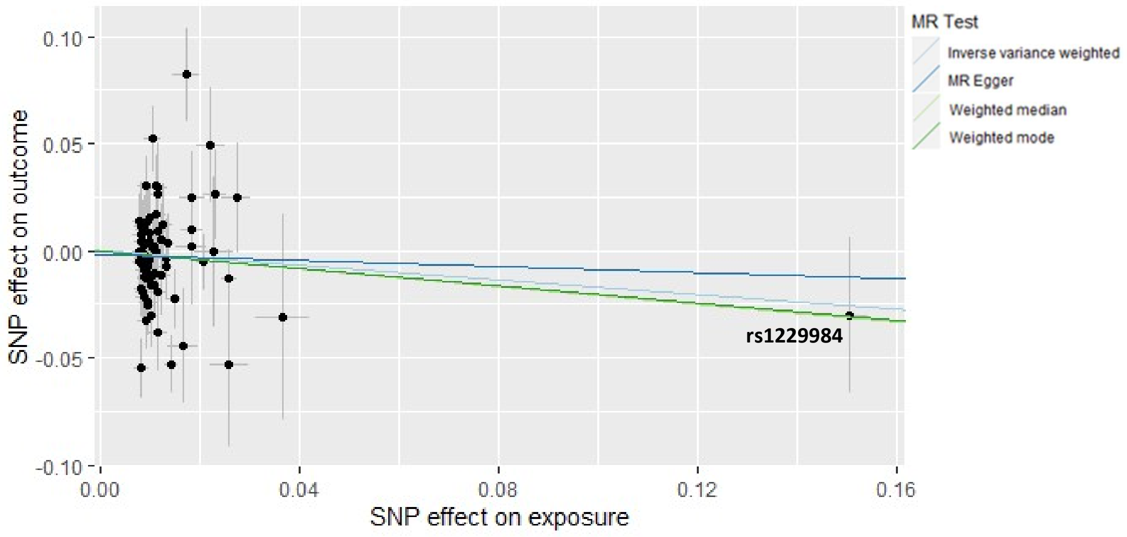

Figure V. Scatter plot of the SNP-alcohol intake (exposure) and SNP-POAG (outcome) association estimates for the full alcohol instrument

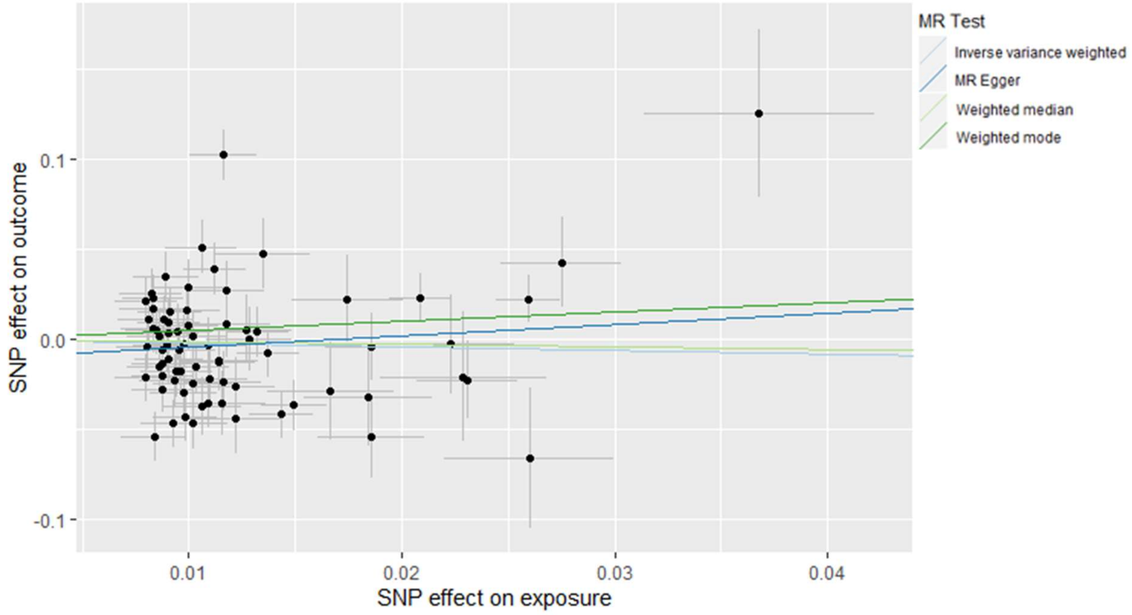

Figure VI. Scatter plot of the SNP-alcohol intake (exposure) and SNP-IOP (outcome) association estimates for the restricted alcohol instrument

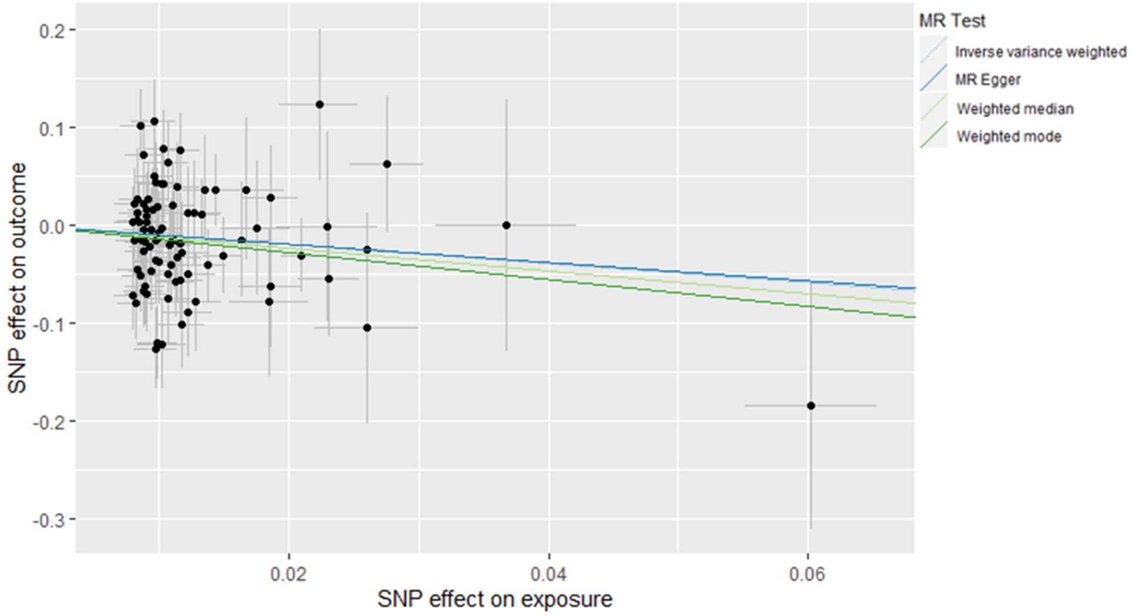

Figure VII. Scatter plot of the SNP-alcohol intake (exposure) and SNP-mRNFL (outcome) association estimates for the restricted alcohol instrument

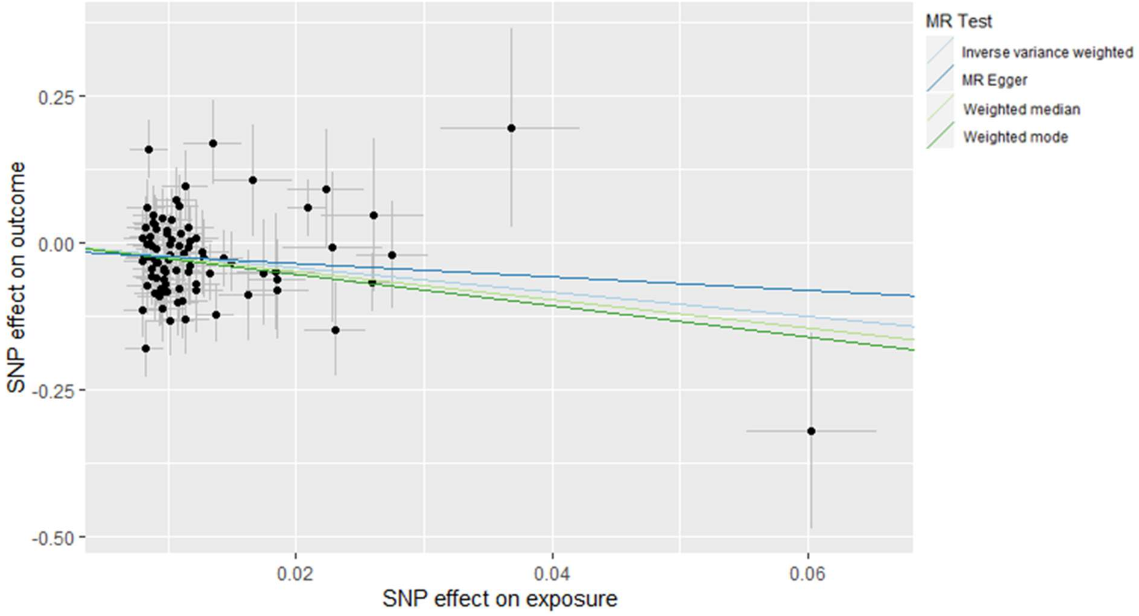

Figure VIII. Scatter plot of the SNP-alcohol intake (exposure) and SNP-mGCIPL (outcome) association estimates for the restricted alcohol instrument

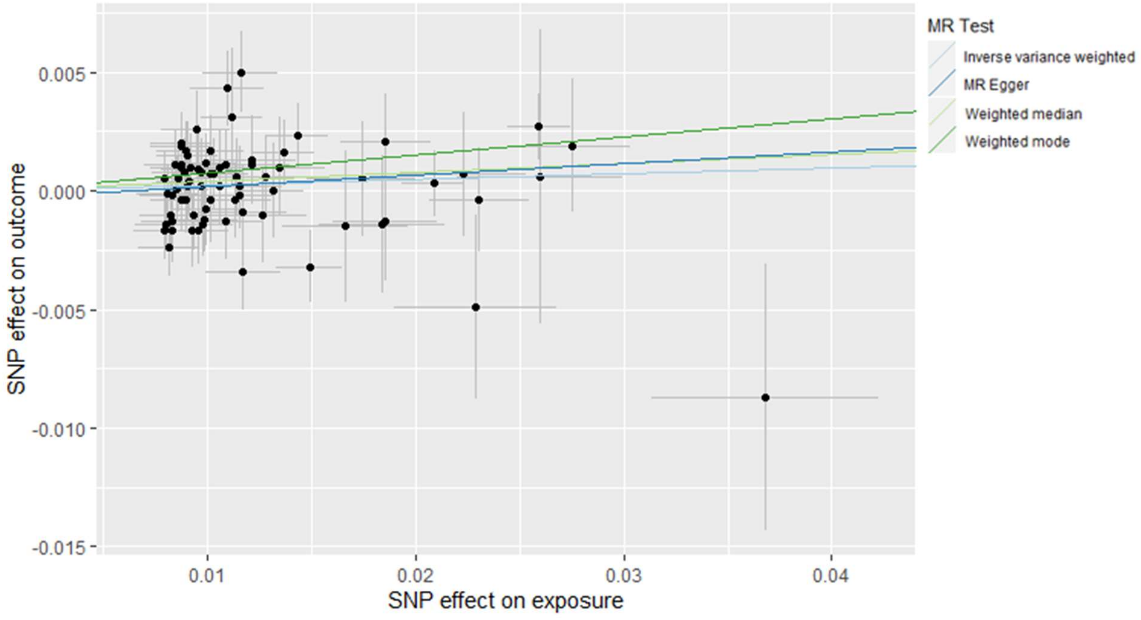

Figure IX. Scatter plot of the SNP-alcohol intake (exposure) and SNP-vCDR (outcome) association estimates for the restricted alcohol instrument

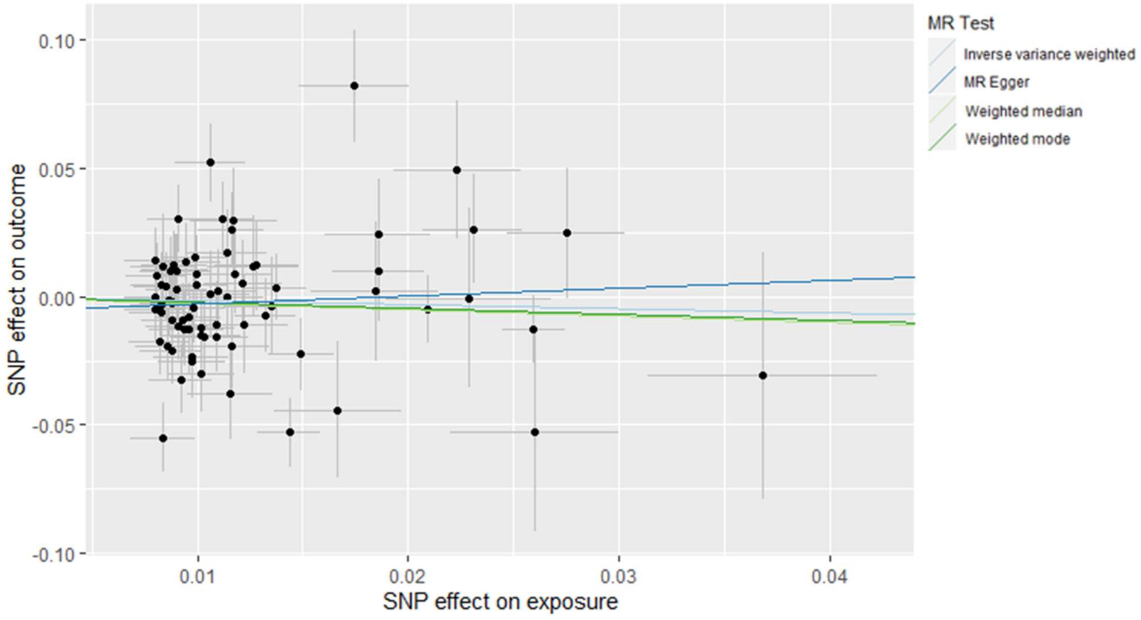

Figure X. Scatter plot of the SNP-alcohol intake (exposure) and SNP-POAG (outcome) association estimates for the restricted alcohol instrument
