# Supplementary material for: The Association of Alcohol Consumption with Glaucoma and Related Traits: Findings from the UK Biobank
Source: Ophthalmol Glaucoma. Author manuscript; Available in PMC 2023 Aug 21. (PMC10239785; doi:10.1016/j.ogla.2022.11.008)
Supplement: Suppl Fig S3 [file NIHMS1876579-supplement-Suppl_Fig_S3.pdf]

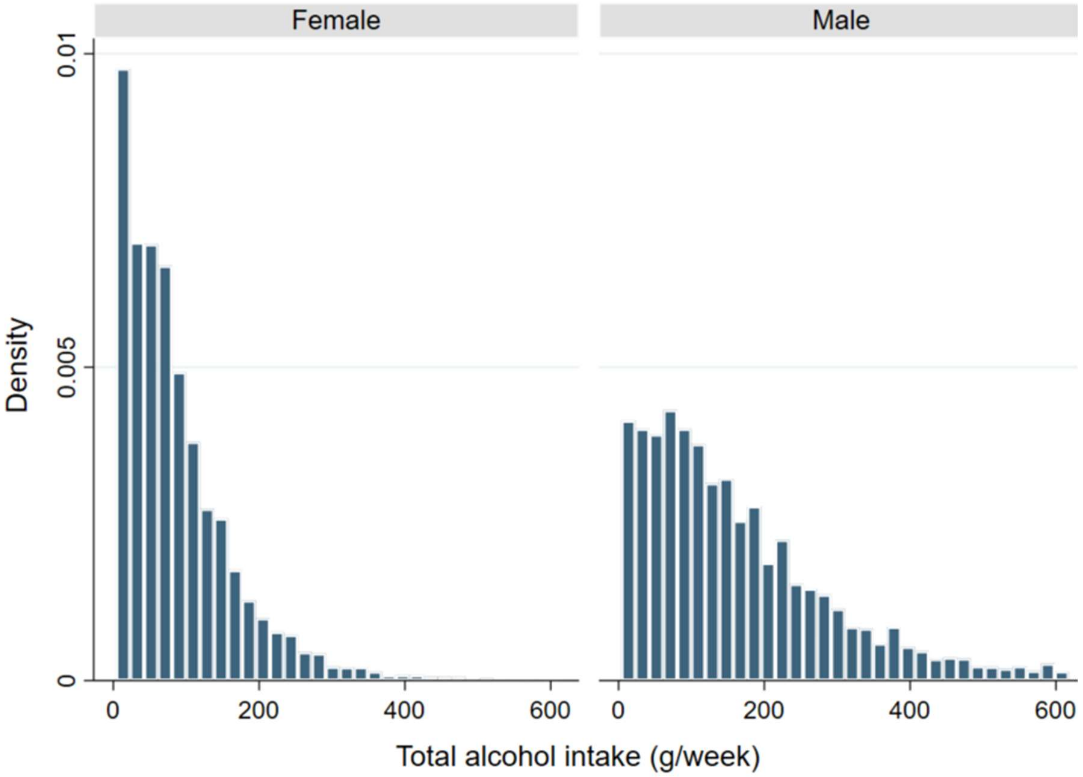

Supplementary Figure S3. Distribution of alcohol intake among regular drinkers in the UK Biobank, stratified by sex
