# Supplementary material for: The Association of Alcohol Consumption with Glaucoma and Related Traits: Findings from the UK Biobank
Source: Ophthalmol Glaucoma. Author manuscript; Available in PMC 2023 Aug 21. (PMC10239785; doi:10.1016/j.ogla.2022.11.008)
Supplement: Suppl Table S5 [file NIHMS1876579-supplement-Suppl_Table_S5.pdf]

Supplementary Table S5. Intraocular pressure, inner retinal OCT measures and glaucoma by alcohol consumption frequency and alcohol intake quintile

	Never	Infrequent	Regular					Former
			Quintile 1	Quintile 2	Quintile 3	Quintile 4	Quintile 5	
IOP (mmHg), mean (SD)	15.87 (3.34)	15.83 (3.41)	15.85 (3.38)	15.97 (3.33)	16.08 (3.35)	16.20 (3.43)	16.46 (3.51)	15.68 (3.39)
mRNFL (µm), mean (SD)	28.67 (3.86)	28.91 (3.90)	29.18 (3.82)	29.20 (3.82)	29.02 (3.85)	28.82 (3.79)	28.52 (3.85)	28.66 (3.87)
mGCIPL (µm), mean (SD)	75.30 (5.14)	75.35 (5.17)	75.42 (5.21)	75.48 (5.25)	75.29 (5.11)	75.12 (5.23)	74.65 (5.33)	75.29 (5.18)
Glaucoma, n (%)	83 (2.04)	164 (1.62)	197 (1.46)	209 (1.54)	202 (1.61)	242 (1.84)	283 (2.15)	77 (2.52)

Notes: Details of alcohol intake quintiles for each cohort are reported in Supplementary Table S2. Summary statistics exclude 1 280, 580 and 1 476 regular drinkers with missing alcohol intake data for IOP, inner retinal OCT measures and glaucoma, respectively.

Abbreviations: OCT, optical coherence tomography; IOP, intraocular pressure; mRNFL, macular retinal nerve fiber layer; mGCIPL, macular ganglion cell–inner plexiform layer; SD, standard deviation.
