# Supplementary material for: The Association of Alcohol Consumption with Glaucoma and Related Traits: Findings from the UK Biobank
Source: Ophthalmol Glaucoma. Author manuscript; Available in PMC 2023 Aug 21. (PMC10239785; doi:10.1016/j.ogla.2022.11.008)
Supplement: Suppl Fig S5 [file NIHMS1876579-supplement-Suppl_Fig_S5.pdf]

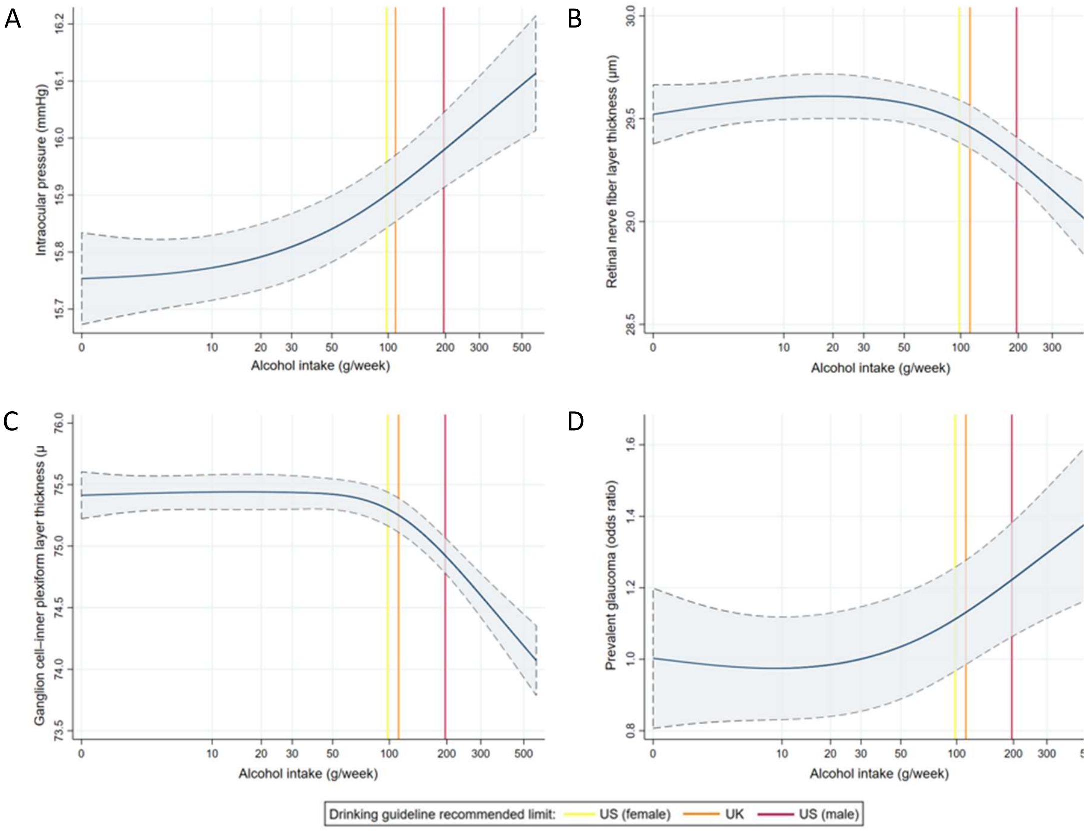

Supplementary Figure S5. Maximally adjusted restricted cubic spline regression models for the association between alcohol intake and **A**) intraocular pressure, **B**) retinal nerve fiber layer thickness, **C**) ganglion cell–inner plexiform layer thickness, and **D**) glaucoma in all participants (excluding former drinkers). Vertical lines represent current UK (112g/week) and US (females 98g/week; males 196g/week) recommended alcohol guidelines.
