# Supplementary material for: The Association of Alcohol Consumption with Glaucoma and Related Traits: Findings from the UK Biobank
Source: Ophthalmol Glaucoma. Author manuscript; Available in PMC 2023 Aug 21. (PMC10239785; doi:10.1016/j.ogla.2022.11.008)
Supplement: Suppl Table S1 [file NIHMS1876579-supplement-Suppl_Table_S1.pdf]

Supplementary Table S1. Details of alcohol category constituents and final portions used to quantify alcohol intake

Alcohol category	Constituents				Final standard portion	
	Description	Alcohol concentration (g/100mL)	Food code	Percentage (%)	Size (mL)	Alcohol concentration (g/100mL)
Red wine	Red wine	9.6	17228	100.0	125.0	9.6
White wine	White wine dry	9.1	17230	25.0	125.0	9.0
	White wine medium	8.9	17231	25.0		
	White wine sparkling	7.6	17232	25.0		
	White wine sweet	10.2	17233	25.0		
Beer/cider	Lager	4.0	17211	35.0	568.3	3.4
	Mild draught	2.5	17215	35.0		
	Cider dry	3.8	17222	15.0		
	Cider sweet	3.7	17224	15.0		
Spirits	Spirits 37.5 vol%	29.6	17246	50.0	28.0	30.7
	Spirits 40 vol%	31.7	17247	50.0		
Fortified wine	Sherry dry	15.7	17235	16.7	62.5	15.4
	Sherry medium	13.3	17236	16.7		
	Sherry sweet	15.6	17237	16.7		
	Port	15.9	17234	50.0		
Other	Cream liqueurs	13.5	17242	33.3	25.0	21.7
	Liqueurs high	31.8	17244	33.3		
	Liqueurs low-medium	19.8	17245	33.3		

Notes: Category constituents, concentrations and food codes are based on those used for the Oxford WebQ.[12-14] Portion sizes are based on those used for the UK Biobank baseline questionnaire.
