# Supplementary material for: The Association of Alcohol Consumption with Glaucoma and Related Traits: Findings from the UK Biobank
Source: Ophthalmol Glaucoma. Author manuscript; Available in PMC 2023 Aug 21. (PMC10239785; doi:10.1016/j.ogla.2022.11.008)
Supplement: Suppl Table S4 [file NIHMS1876579-supplement-Suppl_Table_S4.pdf]

Supplementary Table S4. Participant characteristics by alcohol consumption frequency and alcohol intake quintile (glaucoma cohort; n=84 655)

	Never	Infrequent	Regular					Former
			Quintile 1	Quintile 2	Quintile 3	Quintile 4	Quintile 5	
Sample size	4 077	10 097	13 534	13 544	13 508	13 170	13 189	3 060
Age (years), mean (SD)	55.9 (8.7)	56.6 (8.3)	56.4 (8.2)	56.5 (8.1)	56.7 (8.0)	56.9 (7.9)	57.0 (7.8)	56.7 (8.0)
Sex, n (%)								
Women	2 822 (69.2)	6 980 (69.1)	9 429 (69.7)	8 486 (62.7)	6 550 (52.4)	5 510 (41.8)	2 964 (22.5)	1 601 (52.3)
Men	1 255 (30.8)	3 117 (30.9)	4 105 (30.3)	5 058 (37.3)	5 958 (47.6)	7 660 (58.2)	10 225 (77.5)	1 459 (47.7)
Ethnicity, n (%)								
White	2 273 (55.8)	8 103 (80.3)	12 291 (90.8)	12 679 (93.6)	11 868 (94.9)	12 668 (96.2)	12 787 (97.0)	2 640 (86.3)
Black	392 (9.6)	772 (7.7)	489 (3.6)	343 (2.5)	248 (2.0)	180 (1.4)	112 (0.9)	138 (4.5)
Other	1 412 (34.6)	1 222 (12.1)	754 (5.6)	522 (3.9)	392 (3.1)	322 (2.4)	290 (2.2)	282 (9.2)
Townsend deprivation index, mean (SD)	0.1 (3.3)	-0.4 (3.1)	-1.2 (2.9)	-1.4 (2.8)	-1.4 (2.8)	-1.4 (2.8)`	-1.2 (2.9)	0.0 (3.2)
Body mass index (kg/m^2), mean (SD)	27.8 (5.3)	28.3 (5.6)	27.1 (4.9)	26.6 (4.5)	26.7 (4.4)	27.0 (4.2)	27.7 (4.3)	27.9 (5.4)
Height (cm), mean (SD)	164.1 (9.0)	165.8 (8.8)	166.7 (8.8)	167.9 (9.0)	169.4 (9.0)	170.9 (9.0)	173.6 (8.4)	168.4 (9.1)
Systolic blood pressure (mmHg), mean (SD)	135.9 (18.4)	136.2 (18.5)	135.3 (18.6)	135.5 (18.5)	136.1 (18.0)	137.9 (17.7)	141.6 (17.7)	134.9 (18.0)
Spherical equivalent (D), mean (SD)	-0.3 (2.6)	-0.3 (2.7)	-0.5 (2.9)	-0.5 (2.8)	-0.5 (2.8)	-0.4 (2.8)	-0.3 (2.6)	-0.2 (2.7)
Diabetes, n (%)								
Yes	414 (10.2)	925 (9.2)	664 (4.9)	559 (4.1)	485 (3.9)	525 (4.0)	663 (5.0)	306 (10.0)
Smoking status, n (%)								
Never	3 474 (85.2)	6 542 (64.8)	9 170 (67.8)	8 477 (62.6)	7 235 (57.8)	6 583 (50.0)	5 051 (38.3)	1 429 (46.7)
Previous	449 (11.0)	2 741 (27.2)	3 687 (27.2)	4 517 (33.4)	4 676 (37.4)	5 810 (44.1)	6 735 (51.1)	1 281 (41.9)
Current	154 (3.8)	814 (8.1)	677 (5.0)	550 (4.1)	597 (4.8)	777 (5.9)	1 403 (10.6)	350 (11.4)
Smoking intensity (cigarettes/day), mean (SD)								
Current smokers	14.5 (9.6)	14.8 (7.7)	13.1 (6.7)	13.3 (7.2)	13.2 (7.7)	13.1 (7.4)	15.7 (9.1)	16.6 (9.4)
Physical activity (MET-minutes/week), mean (SD)	2 504 (2 764)	2 690 (2 793)	2 578 (2 570)	2 597 (2 497)	2 657 (2 587)	2 685 (2 647)	2 812 (2 817)	2 738 (2 947)

Notes: Details of alcohol intake quintiles are reported in Supplementary Table S2. Summary statistics exclude 1 476 regular drinkers with missing alcohol intake data.
Abbreviations: SD, standard deviation; D, dioptre; MET, metabolic equivalent of task.
