# Supplementary material for: The Association of Alcohol Consumption with Glaucoma and Related Traits: Findings from the UK Biobank
Source: Ophthalmol Glaucoma. Author manuscript; Available in PMC 2023 Aug 21. (PMC10239785; doi:10.1016/j.ogla.2022.11.008)
Supplement: Suppl Table S3 [file NIHMS1876579-supplement-Suppl_Table_S3.pdf]

Supplementary Table S3. Alcohol intake characteristics by cohort

	Analysis of IOP	Analysis of OCT parameters	Analysis of glaucoma status
**Alcohol consumption frequency, n (%)**			
**Women**	**43 214 (53.1)**	**18 835 (52.1)**	**44 970 (53.1)**
Never	2 703 (6.3)	1 081 (5.7)	2 822 (6.3)
Infrequent	6 707 (15.5)	2 883 (15.3)	6 980 (15.5)
Regular	32 285 (74.7)	14 189 (75.3)	33 567 (74.6)
Red wine drinker	*12 255 (38.0)*	*5 378 (37.9)*	*12 707 (37.9)*
White wine drinker	*9 494 (29.4)*	*4 292 (30.2)*	*9 823 (29.3)*
Beer/cider drinker	*2 686 (8.3)*	*1 182 (8.3)*	*2 794 (8.3)*
Spirits drinker	*2 204 (6.8)*	*902 (6.4)*	*2 286 (6.8)*
Fortified wine drinker	*435 (1.3)*	*179 (1.3)*	*453 (1.3)*
Other drinker	*81 (0.3)*	*33 (0.2)*	*83 (0.2)*
Mixed drinker	*4 577 (14.2)*	*1 976 (13.9)*	*4 699 (14.0)*
Unknown	*913 (2.8)*	*247 (1.7)*	*722 (2.2)*
Former	1 519 (3.5)	682 (3.6)	1 601 (3.6)
**Men**	**38 110 (46.9)**	**17 308 (47.9)**	**39 685 (46.9)**
Never	1 203 (3,2)	455 (2.6)	1 255 (3.2)
Infrequent	2 993 (7.9)	1 301 (7.5)	3 117 (7.9)
Regular	32 518 (85.3)	14 947 (86.4)	33 854 (85.3)
Red wine drinker	*7 771 (23.9)*	*3 597 (24.1)*	*8 095 (23.9)*
White wine drinker	*2 008 (6.2)*	*928 (6.2)*	*2 094 (6.2)*
Beer/cider drinker	*14 224 (43.7)*	*6 505 (43.5)*	*14 774 (43.6)*
Spirits drinker	*1 198 (3.7)*	*492 (3.3)*	*1 229 (3.6)*
Fortified wine drinker	*117 (0.4)*	*54 (0.4)*	*123 (0.4)*
Other drinker	*14 (<0.1)*	*5 (<0.1)*	*14 (<0.1)*
Mixed drinker	*6 381 (19.6)*	*3 002 (20.1)*	*6 535 (19.3)*
Unknown	*911 (2.8)*	*364 (2.4)*	*990 (2.9)*
Former	1 396 (3.7)	605 (3.5)	1 459 (3.7)
**Alcohol intake (g/week), median (range)**			
Quintile 1	18.2 (4.0–34.4)	18.6 (4.4–35.2)	19.2 (4.0–35.2)
Quintile 2	52.9 (34.4–71.7)	55.1 (35.2–72.0)	55.3 (35.2–72.0)
Quintile 3	91.3 (71.7–116.5)	93.6 (72.0–117.6)	92.9 (72.0–116.8)
Quintile 4	151.2 (116.5–196.1)	153.1 (117.6–198.7)	151.9 (116.8–197.0)
Quintile 5	278.2 (196.1–617.4)	279.6 (198.8–608.1)	278.3 (197.1–617.5)

Notes: Alcohol intake quantified in regular drinkers only. Participants were assigned to a particular alcoholic beverage category if they were classified as regular drinkers and >50% of their total alcohol intake was derived from that particular alcoholic beverage type. If no single alcoholic beverage type contributed >50% of a participant's total alcohol intake, they were classified as mixed drinkers.
Abbreviations: IOP, intraocular pressure; OCT, optical coherence tomography; SD, standard deviation.
