# Supplementary material for: The Association of Alcohol Consumption with Glaucoma and Related Traits: Findings from the UK Biobank
Source: Ophthalmol Glaucoma. Author manuscript; Available in PMC 2023 Aug 21. (PMC10239785; doi:10.1016/j.ogla.2022.11.008)
Supplement: Suppl Table S8 [file NIHMS1876579-supplement-Suppl_Table_S8.pdf]

Supplementary Table S8. Association of alcohol consumption frequency with intraocular pressure, inner retinal OCT measures and glaucoma, stratified by sex

Alcohol consumption frequency	Women			Alcohol consumption frequency	Men		
	Estimate	95% CI	P-value		Estimate	95% CI	P-value
**IOP (mmHg)**		(n=43 214)		**IOP (mmHg)**		(n=38 110)	
Never	0.10	(-0.04, 0.25)	0.16	Never	0.04	(-0.20, 0.27)	0.76
Infrequent		Reference		Infrequent		Reference	
Regular	**0.19**	**(0.10, 0.27)**	**<0.001**	Regular	**0.14**	**(0.01, 0.27)**	**0.03**
Former	-0.10	(-0.28, 0.08)	0.26	Former	**-0.22**	**(-0.44, -0.01)**	**0.04**
**mRNFL (µm)**		(n=18 835)		**mRNFL (µm)**		(n=17 308)	
Never	-0.22	(-0.49, 0.04)	0.10	Never	0.28	(-0.12, 0.68)	0.18
Infrequent		Reference		Infrequent		Reference	
Regular	**-0.16**	**(-0.31, 0.00)**	**0.05**	Regular	0.02	(-0.20, 0.23)	0.87
Former	-0.24	(-0.56, 0.07)	0.13	Former	-0.15	(-0.51, 0.21)	0.43
**mGCIPL (µm)**		(n=18 835)		**mGCIPL (µm)**		(n=17 308)	
Never	-0.11	(-0.46, 0.23)	0.53	Never	-0.03	(-0.58, 0.52)	0.91
Infrequent		Reference		Infrequent		Reference	
Regular	-0.09	(-0.29, 0.11)	0.36	Regular	-0.28	(-0.57, 0.02)	0.07
Former	-0.02	(-0.43, 0.39)	0.92	Former	-0.18	(-0.68, 0.31)	0.47
**Glaucoma (%)**		(n=44 970)		**Glaucoma (%)**		(n=39 685)	
Never	1.03	(0.72, 1.46)	0.88	Never	**1.59**	**(1.03, 2.44)**	**0.03**
Infrequent		Reference		Infrequent		Reference	
Regular	1.12	(0.89, 1.39)	0.34	Regular	1.21	(0.92, 1.59)	0.18
Former	**1.71**	**(1.18, 2.49)**	**0.005**	Former	1.44	(0.95, 2.18)	0.09

Notes: Alcohol intake quantified in regular drinkers only. All models adjusted for age, ethnicity, Townsend deprivation index, assessment season, body mass index, height, systolic blood pressure, spherical equivalent, diabetes, smoking status, smoking intensity, physical activity.
Abbreviations: OCT, optical coherence tomography; CI, confidence interval; IOP, intraocular pressure; SD, standard deviation; mRNFL, macular retinal nerve fiber layer; mGCIPL, macular ganglion cell–inner plexiform layer; OR, odds ratio.
