# Supplementary material for: The Association of Alcohol Consumption with Glaucoma and Related Traits: Findings from the UK Biobank
Source: Ophthalmol Glaucoma. Author manuscript; Available in PMC 2023 Aug 21. (PMC10239785; doi:10.1016/j.ogla.2022.11.008)
Supplement: Suppl Table S10 [file NIHMS1876579-supplement-Suppl_Table_S10.pdf]

Supplementary Table S10. Association of alcoholic beverage type with intraocular pressure, inner retinal OCT measures and glaucoma

	IOP (mmHg)			mRNFL (µm)			mGCIPL (µm)			Glaucoma (%)		
	β	95% CI	P-value	β	95% CI	P-value	β	95% CI	P-value	OR	95% CI	P-value
**Alcohol intake from (per SD increase)**												
Red wine (61g/week)	**0.05**	**(0.03, 0.08)**	**<0.001**	**-0.07**	**(-0.11, -0.03)**	**0.001**	**-0.13**	**(-0.18, -0.07)**	**<0.001**	1.04	(0.99, 1.10)	0.13
White wine (48g/week)	**0.06**	**(0.03, 0.08)**	**<0.001**	**-0.07**	**(-0.11, -0.03)**	**<0.001**	**-0.21**	**(-0.27, -0.16)**	**<0.001**	1.02	(0.97, 1.08)	0.42
Beer/cider (91g/week)	0.01	(-0.01, 0.04)	0.27	**-0.11**	**(-0.16, -0.07)**	**<0.001**	**-0.18**	**(-0.24, -0.12)**	**<0.001**	**1.08**	**(1.02, 1.14)**	**0.006**
Spirits (37g/week)	0.01	(-0.01, 0.04)	0.32	-0.03	(-0.07, 0.01)	0.15	**-0.13**	**(-0.18, -0.07)**	**<0.001**	1.04	(0.99, 1.08)	0.10
Fortified wine (10g/week)	0.00	(-0.03, 0.02)	0.78	-0.03	(-0.08, 0.01)	0.10	-0.05	(-0.10, 0.00)	0.08	1.00	(0.96, 1.05)	0.91
Other (3g/week)	0.01	(-0.01, 0.04)	0.37	0.00	(-0.04, 0.05)	0.81	0.00	(-0.04, 0.06)	0.73	1.01	(0.98, 1.05)	0.41

Notes: All models adjusted for age, sex, ethnicity, Townsend deprivation index, assessment season, body mass index, height, systolic blood pressure, spherical equivalent, diabetes, smoking status, smoking intensity, physical activity, and total alcohol intake.
Abbreviations: OCT, optical coherence tomography; IOP, intraocular pressure; mRNFL, macular retinal nerve fiber layer; mGCIPL, macular ganglion cell–inner plexiform layer; β, beta coefficient; CI, confidence interval; OR, odds ratio; SD, standard deviation.
