# Supplementary material for: The Association of Alcohol Consumption with Glaucoma and Related Traits: Findings from the UK Biobank
Source: Ophthalmol Glaucoma. Author manuscript; Available in PMC 2023 Aug 21. (PMC10239785; doi:10.1016/j.ogla.2022.11.008)
Supplement: Suppl Table S9 [file NIHMS1876579-supplement-Suppl_Table_S9.pdf]

Supplementary Table S9. Association of alcohol intake quantity with intraocular pressure, inner retinal OCT measures and glaucoma, stratified by sex

Alcohol intake quantity (g/week)	Women			Alcohol intake quantity (g/week)	Men			$P_{interaction}$
	Estimate	95% CI	P-value		Estimate	95% CI	P-value	
**IOP (mmHg)**		(n=31 767)		**IOP (mmHg)**		(n=31 756)		
Per SD increase	**0.08**	**(0.04, 0.11)**	**<0.001**	Per SD increase	**0.08**	**(0.04, 0.12)**	**<0.001**	0.32
Quintile (range)				Quintile (range)				
Q1 (4.0-23.2)		Reference		Q1 (4.0-53.7)		Reference		
Q2 (23.2-51.9)	0.06	(-0.05; 0.17)	0.29	Q2 (54.0-102.0)	0.09	(-0.02; 0.21)	0.12	
Q3 (52.0-84.0)	0.07	(-0.04; 0.18)	0.20	Q3 (102.1-163.1)	**0.15**	**(0.03; 0.27)**	**0.01**	
Q4 (84.0-137.2)	**0.17**	**(0.06, 0.28)**	**0.002**	Q4 (163.1-255.1)	**0.19**	**(0.07; 0.30)**	**0.002**	
Q5 (137.2-614.5)	**0.20**	**(0.09; 0.31)**	**<0.001**	Q5 (255.1-617.4)	**0.23**	**(0.11; 0.35)**	**<0.001**	
P_{trend}			**<0.001**	P_{trend}			**<0.001**	
**mRNFL (µm)**		(n=13 957)		**mRNFL (µm)**		(n=14 599)		
Per SD increase	**-0.14**	**(-0.20, -0.08)**	**<0.001**	Per SD increase	**-0.18**	**(-0.24, -0.11)**	**<0.001**	0.42
Quintile (range)				Quintile (range)				
Q1 (4.4-23.6)		Reference		Q1 (4.4-54.5)		Reference		
Q2 (23.6-52.9)	-0.05	(-0.25; 0.15)	0.61	Q2 (54.5-101.4)	-0.13	(-0.32; 0.06)	0.19	
Q3 (52.9-84.0)	0.11	(-0.09; 0.31)	0.28	Q3 (101.5-162.5)	**-0.26**	**(-0.45; -0.07)**	**0.007**	
Q4 (84.1-138.4)	-0.05	(-0.25; 0.15)	0.64	Q4 (162.6-254.6)	**-0.37**	**(-0.56; -0.18)**	**<0.001**	
Q5 (138.4-600.1)	**-0.35**	**(-0.55, -0.15)**	**0.001**	Q5 (254.7-608.1)	**-0.54**	**(-0.73; -0.34)**	**<0.001**	
P_{trend}			**0.003**	P_{trend}			**<0.001**	
**mGCIPL (µm)**		(n=13 957)		**mGCIPL (µm)**		(n=14 599)		
Per SD increase	**-0.28**	**(-0.36, -0.19)**	**<0.001**	Per SD increase	**-0.35**	**(-0.44, -0.26)**	**<0.001**	0.31
Quintile (range)				Quintile (range)				
Q1 (4.4-23.6)		Reference		Q1 (4.4-54.5)		Reference		
Q2 (23.6-52.9)	-0.11	(-0.36; 0.15)	0.42	Q2 (54.5-101.4)	-0.21	(-0.47; 0.05)	0.12	
Q3 (52.9-84.0)	-0.02	(-0.28; 0.23)	0.87	Q3 (101.5-162.5)	**-0.41**	**(-0.67; -0.15)**	**0.002**	
Q4 (84.1-138.4)	-0.15	(-0.41; 0.11)	0.25	Q4 (162.6-254.6)	**-0.62**	**(-0.88; -0.36)**	**<0.001**	
Q5 (138.4-600.1)	**-0.70**	**(-0.96; -0.44)**	**<0.001**	Q5 (254.7-608.1)	**-0.99**	**(-1.26; -0.73)**	**<0.001**	
P_{trend}			**<0.001**	P_{trend}			**<0.001**	
**Glaucoma (%)**		(n=32 939)		**Glaucoma (%)**		(n=33 006)		
Per SD increase	**1.11**	**(1.01, 1.21)**	**0.031**	Per SD increase	**1.12**	**(1.04, 1.21)**	**0.002**	0.62
Quintile (range)				Quintile (range)				
Q1 (4.0-23.2)		Reference		Q1 (4.0-54.5)		Reference		
Q2 (23.2-52.8)	1.30	(0.96; 1.76)	0.09	Q2 (54.5-102.2)	1.01	(0.79; 1.30)	0.93	
Q3 (52.9-84.0)	1.28	(0.94; 1.73)	0.12	Q3 (102.3-163.2)	1.04	(0.81; 1.33)	0.77	
Q4 (84.0-137.5)	1.11	(0.80; 1.53)	0.53	Q4 (163.2-255.3)	1.25	(0.98; 1.60)	0.07	
Q5 (137.5-616.1)	**1.47**	**(1.08, 2.00)**	**0.01**	Q5 (255.3-617.5)	**1.34**	**(1.05; 1.72)**	**0.02**	
P_{trend}			0.08	P_{trend}			**0.004**	

Notes: Alcohol intake quantified in regular drinkers only. All models adjusted for age, ethnicity, Townsend deprivation index, assessment season, body mass index, height, systolic blood pressure, spherical equivalent, diabetes, smoking status, smoking intensity, physical activity.
Abbreviations: OCT, optical coherence tomography; CI, confidence interval; IOP, intraocular pressure; SD, standard deviation; mRNFL, macular retinal nerve fiber layer; mGCIPL, macular ganglion cell–inner plexiform layer; OR, odds ratio; SD, standard deviation.
