# Supplementary material for: The Association of Alcohol Consumption with Glaucoma and Related Traits: Findings from the UK Biobank
Source: Ophthalmol Glaucoma. Author manuscript; Available in PMC 2023 Aug 21. (PMC10239785; doi:10.1016/j.ogla.2022.11.008)
Supplement: Suppl Table S11 [file NIHMS1876579-supplement-Suppl_Table_S11.pdf]

Supplementary Table S11. Association of alcohol consumption frequency and alcohol intake quantity with intraocular pressure and glaucoma (alternate definitions)

	IOPg (mmHg)			IOPcc without imputation (mmHg)			Glaucoma (self-report + ICD POAG)			Glaucoma (self-report + ICD POAG/unspecified)		
	β	95% CI	P-value	β	95% CI	P-value	OR	95% CI	P-value	OR	95% CI	P-value
**Alcohol consumption**												
Never	0.04	(-0.09, 0.17)	0.56	0.08	(-0.03, 0.20)	0.17	1.23	(0.94, 1.62)	0.14	1.25	(0.95, 1.64)	0.11
Infrequent		Reference			Reference			Reference			Reference	
Regular	**0.22**	**(0.15, 0.30)**	**<0.001**	**0.16**	**(0.09, 0.22)**	**<0.001**	1.11	(0.94, 1.32)	0.22	1.13	(0.95, 1.34)	0.17
Former	-0.06	(-0.20, 0.08)	0.42	**-0.17**	**(-0.30, -0.04)**	**0.01**	**1.51**	**(1.14, 1.99)**	**0.004**	**1.51**	**(1.15, 2.00)**	**0.004**
**Alcohol intake (g/week)**												
Per SD increase	**0.18**	**(0.15, 0.21)**	**<0.001**	**0.08**	**(0.04, 0.10)**	**<0.001**	**1.11**	**(1.05, 1.18)**	**0.001**	**1.11**	**(1.04, 1.18)**	**0.001**
Quintiles												
Quintile 1		Reference			Reference			Reference			Reference	
Quintile 2	**0.15**	**(0.07, 0.23)**	**<0.001**	**0.09**	**(0.01, 0.17)**	**0.02**	1.09	(0.89, 1.33)	0.41	1.07	(0.88, 1.31)	0.50
Quintile 3	**0.26**	**(0.17, 0.34)**	**<0.001**	**0.15**	**(0.07, 0.23)**	**<0.001**	1.11	(0.91, 1.37)	0.30	1.08	(0.89, 1.33)	0.44
Quintile 4	**0.32**	**(0.24, 0.41)**	**<0.001**	**0.16**	**(0.08, 0.24)**	**<0.001**	**1.24**	**(1.02, 1.51)**	**0.04**	1.20	(0.99, 1.46)	0.07
Quintile 5	**0.53**	**(0.44, 0.62)**	**<0.001**	**0.24**	**(0.16, 0.33)**	**<0.001**	**1.38**	**(1.13, 1.68)**	**0.002**	**1.35**	**(1.11, 1.65)**	**0.003**
P_{trend}			**<0.001**			**<0.001**			**0.001**			**0.002**

Notes: Alcohol intake quantified in regular drinkers only. Details of alcohol intake quintiles for each cohort are reported in Supplementary Table S2. All models adjusted for age, sex, ethnicity, Townsend deprivation index, assessment season, body mass index, height, systolic blood pressure, spherical equivalent, diabetes, smoking status, smoking intensity, physical activity.

Abbreviations: IOPg, Goldmann-correlated intraocular pressure; IOPcc, corneal-compensated intraocular pressure; ICD, International Classification of Diseases; POAG, primary open-angle glaucoma; β, beta-coefficient; CI, confidence interval; OR, odds ratio.
