# Supplementary material for: The Association of Alcohol Consumption with Glaucoma and Related Traits: Findings from the UK Biobank
Source: Ophthalmol Glaucoma. Author manuscript; Available in PMC 2023 Aug 21. (PMC10239785; doi:10.1016/j.ogla.2022.11.008)
Supplement: Suppl Table S7 [file NIHMS1876579-supplement-Suppl_Table_S7.pdf]

Supplementary Table S7. Details and model selection for restricted cubic spline regression analyses

Number of knots	Knot positions (quantiles)	Akaike Information Criterion (AIC)			
		IOP	mRNFL	mGCIPL	Glaucoma
3	0.1; 0.5; 0.9	**330542.1**	**154324.8**	**169957.0**	**10933.4**
4	0.05; 0.35; 0.65; 0.95	330543.4	154327.3	169958.5	10933.4
5	0.05; 0.275; 0.5; 0.725; 0.95	330544.9	154327.8	169960.5	10933.5
6	0.05; 0.23; 0.41; 0;59; 0.77; 0.95	330546.3	154328.9	169962.4	10935.2
7	0.025; 0.1833; 0.3417; 0.5; 0.6583; 0.8167; 0.975	330547.9	154350.5	169963.3	10937.1

Notes: All models adjusted for age, sex, ethnicity, Townsend deprivation index, assessment season, body mass index, height, systolic blood pressure, spherical equivalent, diabetes, smoking status, smoking intensity, physical activity. Values in bold indicate the final selected model. For models with equal AIC, the simpler model (fewer knots) was selected.
Abbreviations: IOP, intraocular pressure; mRNFL, macular retinal nerve fiber layer; mGCIPL, macular ganglion cell–inner plexiform layer.
