# Supplementary material for: The Association of Alcohol Consumption with Glaucoma and Related Traits: Findings from the UK Biobank
Source: Ophthalmol Glaucoma. Author manuscript; Available in PMC 2023 Aug 21. (PMC10239785; doi:10.1016/j.ogla.2022.11.008)
Supplement: Suppl Table S12 [file NIHMS1876579-supplement-Suppl_Table_S12.pdf]

Supplementary Table S12. Association of alcohol consumption frequency and alcohol intake quantity with intraocular pressure, inner retinal OCT measures and glaucoma, with further adjustment for additional covariables

	IOP (mmHg)			mRNFL (µm)			mGCIPL (µm)			Glaucoma (%)		
	β	95% CI	P-value	β	95% CI	P-value	β	95% CI	P-value	OR	95% CI	P-value
**Alcohol consumption**		(n = 50,895)			(n = 22,858)			(n = 22,858)			(n = 52,800)	
Never	0.01	(-0.16, 0.19)	0.88	-0.05	(-0.36, 0.25)	0.73	0.00	(-0.41, 0.40)	0.99	1.01	(0.67, 1.54)	0.94
Infrequent		Reference			Reference			Reference			Reference	
Regular	**0.18**	**(0.08, 0.27)**	**<0.001**	-0.15	(-0.32, 0.01)	0.07	-0.19	(-0.41, 0.02)	0.08	1.07	(0.85, 1.34)	0.59
Former	-0.07	(-0.25, 0.11)	0.43	-0.27	(-0.58, 0.03)	0.08	0.00	(-0.41, 0.40)	0.99	1.40	(0.95, 2.05)	0.09
**Alcohol intake (g/week)**		(n = 41,198)			(n = 18,617)			(n = 18,617)			(n = 42,636)	
Per SD increase	**0.10**	**(0.07, 0.14)**	**<0.001**	**-0.16**	**(-0.22, -0.10)**	**<0.001**	**-0.36**	**(-0.44, -0.28)**	**<0.001**	**1.12**	**(1.05, 1.22)**	**0.002**
Quintiles												
Quintile 1		Reference			Reference			Reference			Reference	
Quintile 2	0.02	(-0.08, 0.12)	0.67	-0.03	(-0.20, 0.14)	0.70	0.01	(-0.21, 0.24)	0.88	1.03	(0.80, 1.32)	0.83
Quintile 3	**0.10**	**(0.00, 0.20)**	**0.05**	-0.17	(-0.34, 0.00)	0.06	**-0.30**	**(-0.53, -0.07)**	**0.01**	1.14	(0.89, 1.47)	0.30
Quintile 4	**0.16**	**(0.05, 0.26)**	**0.003**	**-0.25**	**(-0.43, -0.08)**	**0.005**	**-0.43**	**(-0.66, -0.20)**	**<0.001**	1.16	(0.90, 1.49)	0.26
Quintile 5	**0.28**	**(0.17, 0.38)**	**<0.001**	**-0.44**	**(-0.62, -0.25)**	**<0.001**	**-0.94**	**(-1.19, -0.70)**	**<0.001**	**1.37**	**(1.06, 1.76)**	**0.02**
P_{trend}			**<0.001**			**<0.001**			**<0.001**			**0.01**

Notes: Alcohol intake quantified in regular drinkers only. Details of alcohol intake quintiles for each cohort are reported in Supplementary Table S2. All models adjusted for age, sex, ethnicity, Townsend deprivation index, assessment season, body mass index, height, systolic blood pressure, spherical equivalent, diabetes, smoking status, smoking intensity, physical activity, total caffeine intake, total cholesterol, statin use, oral beta blocker use.
Abbreviations: OCT, optical coherence tomography; IOP, intraocular pressure; mRNFL, macular retinal nerve fiber layer; mGCIPL, macular ganglion cell–inner plexiform layer; β, beta coefficient; CI, confidence interval; OR, odds ratio; SD, standard deviation.
